# Gene-teratogen interactions influence the penetrance of birth defects by altering Hedgehog signaling strength

**DOI:** 10.1242/dev.199867

**Published:** 2021-10-04

**Authors:** Jennifer H. Kong, Cullen B. Young, Ganesh V. Pusapati, F. Hernán Espinoza, Chandni B. Patel, Francis Beckert, Sebastian Ho, Bhaven B. Patel, George C. Gabriel, L. Aravind, J. Fernando Bazan, Teresa M. Gunn, Cecilia W. Lo, Rajat Rohatgi

**Affiliations:** 1 Departments of Biochemistry and Medicine, Stanford University School of Medicine, Stanford, CA 94305, USA; 2 Department of Developmental Biology, University of Pittsburgh School of Medicine, Pittsburgh, PA 15201, USA; 3 National Center for Biotechnology Information, National Library of Medicine, National Institutes of Health, Bethesda, MD 20894, USA; 4 H Bioconsulting, Stillwater, MN 50082, USA; 5 McLaughlin Research Institute, Great Falls, MT 59405, USA

**Keywords:** Hedgehog signaling, Smoothened, Gene-environment interactions, Left-right patterning, Morphogen, Structural birth defects

## Abstract

Birth defects result from interactions between genetic and environmental factors, but the mechanisms remain poorly understood. We find that mutations and teratogens interact in predictable ways to cause birth defects by changing target cell sensitivity to Hedgehog (Hh) ligands. These interactions converge on a membrane protein complex, the MMM complex, that promotes degradation of the Hh transducer Smoothened (SMO). Deficiency of the MMM component MOSMO results in elevated SMO and increased Hh signaling, causing multiple birth defects. *In utero* exposure to a teratogen that directly inhibits SMO reduces the penetrance and expressivity of birth defects in *Mosmo^−/−^* embryos. Additionally, tissues that develop normally in *Mosmo^−/−^* embryos are refractory to the teratogen. Thus, changes in the abundance of the protein target of a teratogen can change birth defect outcomes by quantitative shifts in Hh signaling. Consequently, small molecules that re-calibrate signaling strength could be harnessed to rescue structural birth defects.

## Abstract (precis)

**Summary:** The MMM complex is a module that attenuates Hedgehog signaling, regulating left-right asymmetry of organs as well as patterning and morphogenesis of multiple tissues in the embryo.

## INTRODUCTION

Six percent of newborns suffer from structural birth defects, leading to 8 million cases per year worldwide (Christianson et al., 2005). Many of these structural defects require surgical intervention early in life and lead to adverse long-term health consequences. The underlying mechanisms driving birth defects remain unknown in a majority of cases. Complex interactions between genetic and environmental factors are thought to shift morphogen signaling beyond the threshold required for normal developmental patterning (Beames and Lipinski, 2020; Finnell, 1999; Krauss and Hong, 2016). However, in most cases the specific molecular mechanisms remain poorly understood. Penetrance and expressivity of birth defects, both between embryos and between tissues, remains unpredictable and confounds identification of causal factors. Improved understanding of molecular mechanisms is crucial to developing strategies to alleviate the significant public health burden of birth defects.

The Hedgehog (Hh) pathway is one of a handful of signaling systems that regulate developmental patterning and morphogenesis of many tissues, including the face, limbs, heart, lungs, brain and spinal cord (McMahon et al., 2003). Developing tissues are often exquisitely sensitive to the precise amplitude of Hh signaling. Even small changes in signaling strength can cause birth defects in mice and humans (Nieuwenhuis and Hui, 2005). Hh ligands are considered to be classical morphogens: secreted molecules that direct cell-fate choices in a dose-dependent manner (Lee et al., 2016). Temporal and spatial gradients of Hh ligands are translated into intracellular gradients of activity of the GLI transcription factors in target cells (Harfe et al., 2004; Jacob and Briscoe, 2003; Stamataki et al., 2005). Varying Hh signaling strength leads target cells to adopt different cell fates (Dessaud et al., 2008). Given the centrality of morphogen gradients in developmental patterning, considerable research effort has focused on understanding how they are established in tissues. However, this ligand-centric view of patterning is incomplete. Specific signaling mechanisms function in target cells to regulate their sensitivity to morphogens. Indeed, cell fate decisions often depend on both the extracellular concentration of ligands and the reception sensitivity of target cells to these ligands. A prominent example of such a mechanism can be found in the WNT pathway: cell-surface levels of frizzled receptors (and consequently WNT sensitivity) is controlled by the transmembrane E3 ubiquitin ligases ZNRF3 and RNF43 (de Lau et al., 2014). These E3 ligases are themselves controlled by R-spondins, secreted ligands that play central roles in both pattern formation during development and in postnatal tissue homeostasis.

Our analysis of a gene called *Mosmo* (modulator of Smoothened) led us to uncover a cell-surface pathway that regulates the sensitivity to Hh ligands, and consequently the development of multiple tissues. Mouse genetic analysis revealed that *Mosmo* uniquely functions to tune the Hh signaling gradient in target cells by promoting the degradation of Smoothened (SMO), a 7-pass transmembrane protein that carries the Hh signal across the plasma membrane. Interestingly, mutations in *Mosmo* (which increase SMO protein abundance) influence penetrance, expressivity and tissue specificity of birth defects caused by an exogenous teratogen that directly inhibits SMO activity. These findings show that the penetrance of birth defects can be modulated by gene-environment interactions that alter ligand sensitivity in the Hh pathway.

## RESULTS

### MOSMO is required for embryonic development

**Fig. 1.**
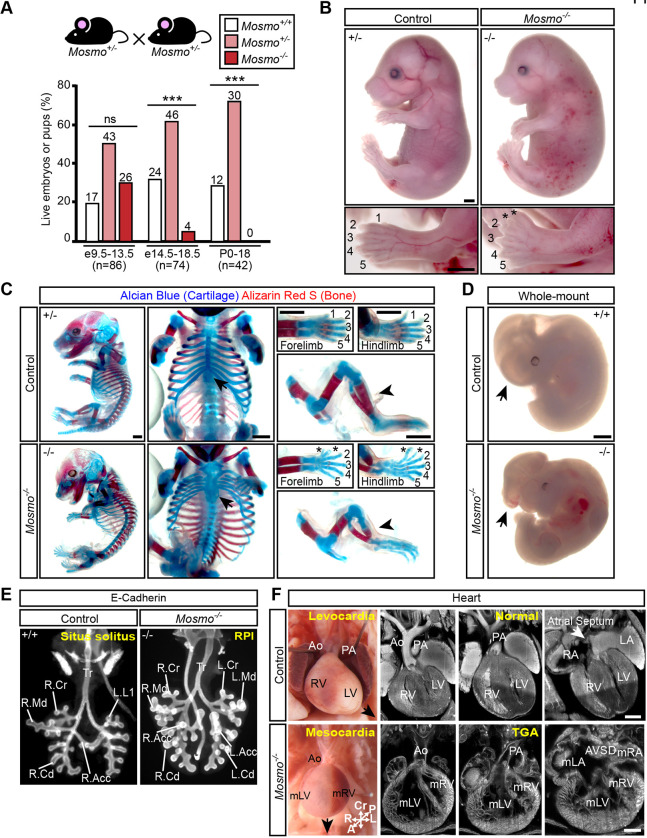
**Loss of *Mosmo* results in embryonic lethality and developmental defects.** (A) Viability of offspring derived from *Mosmo^+/−^*×*Mosmo^+/−^* crosses at the indicated developmental stages. Statistical significance was determined by the chi-squared test (ns>0.05, ***P<0.001) and *n*=number of live embryos collected. See Table S1 for full details. (B) Whole-mount images of E16.5 control (*Mosmo^+/−^*) and *Mosmo^−/−^* littermates show that the latter have edema and preaxial polydactyly. See Table S2 for a detailed list of phenotypes in each embryo analyzed. Scale bars: 1 mm. (C) Skeletons from E16.5 control (*Mosmo^+/−^*) and *Mosmo^−/−^* littermates stained with Alcian Blue and Alizarin Red S to visualize cartilage and calcified bone, respectively. Polydactyly (asterisks), sternal clefting (middle column, arrows) and tibial truncation (tibial hemimelia, right column, arrowheads) were observed in *Mosmo^−/−^* embryos. Scale bars: 1 mm. (D) Whole-mount images of E11.5 control (*Mosmo^+/+^*) and *Mosmo^−/−^* littermates show that the latter suffer from exencephaly (arrow). Scale bar: 1 mm. (E) Whole-mount lungs (ventral view) of E12.5 control (*Mosmo^+/+^*) and *Mosmo*^−/−^ embryos immunostained for E-cadherin to show the airway epithelium and allow for detailed branching analysis. Normal mouse lungs have one lobe on the left (L.L1) and four lobes on the right (R.Acc, right accessory; R.Cd, right caudal; R.Cr, right cranial; R.Md, right middle). *Mosmo*^−/−^ lungs exhibit right pulmonary isomerism (RPI), a duplication of the right lung morphology on the left side (L.Acc, left accessory; L.Cd, left caudal; L.Cr, left cranial; L.Md, left middle). Further details are provided in Table S3. (F) *Mosmo^−/−^* embryos exhibit complex CHDs associated with abnormal left-right patterning of the heart. Necropsy and episcopic confocal fluorescence microscopy (ECM) images of representative embryonic hearts from E16.5 *Mosmo^+/+^* (control, top) and *Mosmo*^−/−^ embryos (bottom). The *Mosmo*^−/−^ heart has no apparent apex (black arrow), indicating mesocardia, with the aorta (Ao) abnormally positioned anteriorly. The Ao is situated anterior to the pulmonary artery (PA) and inserted into the morphological right ventricle (mRV) situated on the left of the body, while the pulmonary artery (PA) emerges from the morphological left ventricle (mLV) positioned on the right of the body, findings that are diagnostic of transposition of the great arteries (TGAs). Other findings include non-compaction of the ventricular myocardium and an unbalanced atrioventricular septal defect (AVSD) with symmetric insertions of both inferior and superior vena cava suggesting right atrial isomerism (mRA). Scale bars: 0.5 mm. A detailed list of cardiac phenotypes observed in each embryo can be found in Table S2.

*Mosmo* (previously named *Atthog*) is a previously unannotated gene we initially identified in a loss-of-function CRISPR screen conducted in NIH/3T3 fibroblasts designed to find negative attenuators of Hh signaling (Pusapati et al., 2018). *Mosmo* encodes an 18.2 kDa four-pass transmembrane (4TM) protein that defines a distinct branch of a large superfamily of eukaryotic 4TM proteins that include the claudins, which are known to function at tight junctions. Depletion of MOSMO in cultured cells results in increased accumulation of the Hh transducer SMO on the plasma membrane and the primary cilium membrane, resulting in hyper-responsiveness to Hh ligands. *Mosmo* is widely expressed in mouse embryos, based on *in situ* hybridization (Fig. S1A) and the analysis of published single-cell RNAseq data (Pijuan-Sala et al., 2019) (Fig. S1B). To understand the developmental roles of MOSMO, we used CRISPR/Cas9 genome editing to generate mice carrying null alleles of *Mosmo* (Fig. S2A and S2B). Although *Mosmo*^+/−^ mice developed normally, no live *Mosmo*^−/−^ pups were recovered from heterozygous intercrosses (Fig. 1A and Table S1). Most *Mosmo*^−/−^ embryos die by gestational day 14.5 (E14.5) (Fig. 1A and Table S1). We conclude that the function of MOSMO is essential for embryonic development.

### *Mosmo* is required for proper left-right patterning and heart, limb and lung development

*Mosmo* deficiency results in developmental defects across many organ systems. *Mosmo*^−/−^ embryos have preaxial polydactyly in both forelimbs and hindlimbs (Fig. 1B,C). Whole-mount skeletal staining revealed additional skeletal defects, including a split sternum (Fig. 1C, arrows) and truncated tibia (Fig. 1C, arrowheads). A subset of *Mosmo*^−/−^ embryos exhibited exencephaly (Fig. 1D). Detailed necropsy examination of the internal anatomy revealed that all *Mosmo*^−/−^ embryos had heterotaxy: discordant patterning of the left-right body axis manifested as abnormalities in lung lobation and abnormal left-right positioning of multiple visceral organs, including the heart, stomach, spleen and pancreas (Table S2). Analysis of the early lung branching pattern indicated that most *Mosmo*^−/−^ embryos have either complete or partial right pulmonary isomerism: a duplication of the right lung morphology on the left side (Fig. 1E and Fig. S3A, Table S3). Analysis of the developing heart using episcopic confocal fluorescence microscopy (ECM) revealed that all *Mosmo*^−/−^ embryos have complex congenital heart defects (CHDs). The most common CHDs observed are transposition of the great arteries (TGA) and atrioventricular septal defects (AVSDs) (Fig. 1F and Table S2). TGA and AVSDs are classified as ‘critical’ heart defects in humans as they require surgical intervention soon after birth. *Mosmo*^−/−^ embryos likely die *in utero* due to these complex structural heart defects.

### *Mosmo^−/−^* phenotypes are correlated with elevated Hh signaling activity

**Fig. 2.**
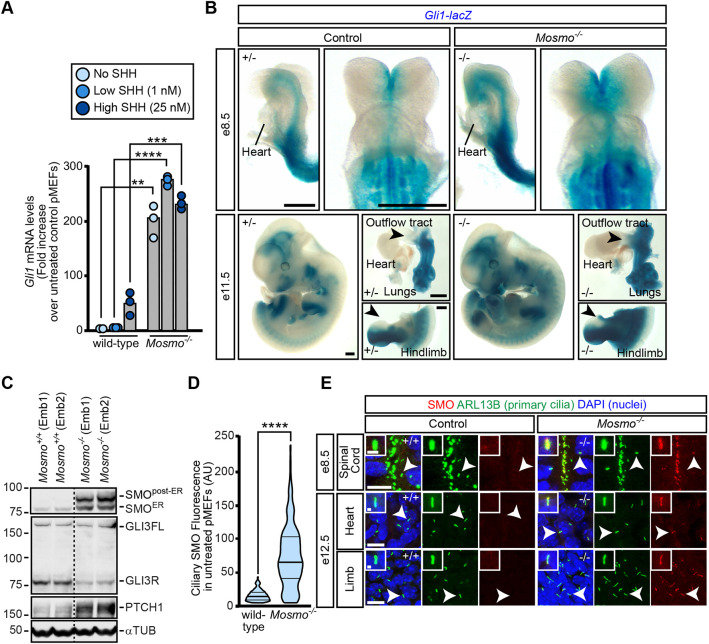
**Loss of *Mosmo* results in elevated Hh signaling activity.** Hh signaling activity in primary mouse embryonic fibroblasts (pMEFs) and embryonic tissues was assessed using expression of *Gli1*, a direct Hh target gene, or accumulation of SMO in primary cilia. (A) *Gli1* mRNA abundance in wild-type (*Mosmo^+/+^*) and *Mosmo^−/−^* pMEFs was measured using qRT-PCR. Data are the median *Gli1* mRNA values derived from the two or three individual measurements shown as circles. The statistical analysis between the two groups was determined using an unpaired two-tailed *t*-test (**P<0.01,***P<0.001 and ****P<0.0001). (B) X-gal staining was used to visualize *Gli1-lacZ* expression in whole-mount preparations of mouse embryos. Arrowheads indicate areas of elevated Hh signaling activity in the cardiac outflow tract and anterior hindlimb. Scale bars: 0.5 mm. (C) Immunoblot used to measure protein abundance of SMO, GLI3, PTCH1 and ɑTUB (a loading control) in whole-embryo lysates prepared from E12.5 wild-type (*Mosmo^+/+^*) and *Mosmo^−/−^* littermates. (D,E) Immunofluorescence (IF) was used to measure SMO abundance (red) in primary cilia (ARL13B, green) in pMEFs (D) and various embryonic tissues (E). Violin plots in D summarize ciliary SMO fluorescence data from wild-type (*n*=108) and *Mosmo^−/−^* (*n*=156) pMEFs, with horizontal lines indicating the median and interquartile range. Statistical significance was determined using a two-tailed Mann–Whitney test (****P<0.0001). (E) DAPI (blue) marks nuclei. White arrowheads indicate the primary cilium enlarged in the inset. Scale bars: 10 µm in merged panels; 1 µm in insets.

To understand the etiology of the birth defects observed in *Mosmo*^−/−^ embryos, we focused on the Hh signaling pathway because *Mosmo* was originally identified as an attenuator of Hh signaling in our CRISPR screens (Pusapati et al., 2018), and many of the *Mosmo*^−/−^ phenotypes (i.e. polydactyly and exencephaly) can be caused by elevated Hh signaling (Hui and Joyner, 1993). To assess Hh signaling activity in *Mosmo^−/−^* cells, primary mouse embryonic fibroblasts (pMEFs) were isolated and treated with varying concentrations of Sonic hedgehog (SHH), a secreted ligand that initiates Hh signaling in target cells. Compared with cells from wild-type littermate controls, Hh signaling was elevated in *Mosmo^−/−^* pMEFs (Fig. 2A). A low concentration of SHH (1 nM) that failed to fully activate expression of the Hh target gene *Gli1* in wild-type pMEFs was sufficient to maximally activate *Gli1* in *Mosmo*^−/−^ pMEFs.

To assess levels of Hh signaling activity in *Mosmo^−/−^* embryos, we crossed *Mosmo^+/−^* mice with *Gli1^lacZ/+^* mice, an extensively used Hh reporter line in which a lacZ transgene (encoding β-galactosidase) was inserted into the first coding exon of *Gli1* (Bai et al., 2002). The *Gli1-lacZ* expression pattern recapitulated endogenous *Gli1* expression and the expression of other Hh target genes such as *Ptch1* (Goodrich et al., 1997; Guzzetta et al., 2020; Hui et al., 1994). At E8.5 and E9.5, *Gli1-lacZ* was expressed in the neural tube, somites and secondary heart field (SHF) (Fig. 2B and Fig. S3B) (Guzzetta et al., 2020). At E11.5, *Gli1* reporter activity was observed in the brain (telencephalon and diencephalon), spinal cord, limbs, lungs, frontonasal processes and pharyngeal endoderm (Fig. 2B and Fig. S3B). Compared with littermate controls (*Mosmo^+/+^* and *Mosmo^+/−^*), *Gli1-lacZ* expression was elevated in *Mosmo^−/−^* embryos at all embryonic ages analyzed. The expansion of *Gli1* expression is notable in the developing limb and cardiac outflow tract (Fig. 2B and Fig. S3C), consistent with the finding of polydactyly and outflow tract-related heart defects in *Mosmo^−/−^* embryos (Table S2) (Goddeeris et al., 2007). Consistent with the elevation of *Gli1* expression, we observed an increase in PTCH1 (encoded by a direct Hh target gene) and a decrease in GLI3R (the major Hh transcriptional repressor) in *Mosmo^−/−^* whole embryo lysates, showing that *Mosmo* deficiency results in increased Hh signaling activity *in vivo* (Fig. 2C).

Hh signal transmission across the plasma membrane requires the SHH-induced accumulation of SMO in the membrane of the primary cilium (Corbit et al., 2005; Rohatgi et al., 2007). The loss of *Mosmo* led to the constitutive, high-level accumulation of SMO in the ciliary membrane in pMEFs (Fig. 2D) and all embryonic tissues analyzed (Fig. 2E). We also observed an increase in SMO protein abundance in *Mosmo^−/−^* embryo lysates (Fig. 2C). This increase was dramatic for the SMO protein band that migrated more slowly in the SDS-PAGE gel, which represents the population of SMO that has transversed the endoplasmic reticulum (post-ER) and acquired glycan modifications attached in the Golgi. We conclude that *Mosmo* functions to attenuate Hh signaling strength both in cells and embryos by reducing SMO levels in the ciliary membrane. Re-expression of MOSMO into *Mosmo^−/−^* cells restored both wild-type Hh signaling and ciliary SMO levels (Fig. S4A,B).

### MOSMO interacts with MEGF8 and MGRN1 to form the MMM complex

The cellular phenotypes (elevated ciliary SMO and sensitivity to SHH) and developmental defects (polydactyly, heterotaxy and CHDs) seen in *Mosmo^−/−^* embryos were reminiscent of those caused by the loss of either *Megf8* or *Mgrn1* and *Rnf157*, components of a membrane-tethered E3 ubiquitin ligase complex that ubiquitylates SMO and accelerates its endocytosis and degradation (Kong et al., 2020) (Table 1 and Fig. S4C, Table S2). To determine whether MEGF8 and MOSMO are part of the same pathway, we compared Hh signaling activity and SMO abundance in cells lacking each gene individually (*Mosmo^−/−^* and *Megf8^−/−^* single knockouts) to cells lacking both (*Mosmo*^−/−^;*Megf8*^−/−^ double knockouts). The increase in cell-surface SMO and SHH sensitivity seen in *Mosmo*^−/−^;*Megf8*^−/−^ double knockout cells was comparable with that seen in *Mosmo^−/−^* and *Megf8*^−/−^ single knockout cells (Fig. S4D). Similarly, the birth defects observed in *Mosmo^−/−^*;*Megf8^m/m^* double mutant mouse embryos were comparable in both penetrance and expressivity to *Mosmo^−/−^* and *Megf8^m/m^* single mutant embryos (Table 1 and Table S2). Taken together, analysis in both cells and embryos suggested that *Mosmo* and *Megf8* belong to the same epistasis group, and thus the proteins encoded by these genes likely belong to the same pathway.

**Table 1.**
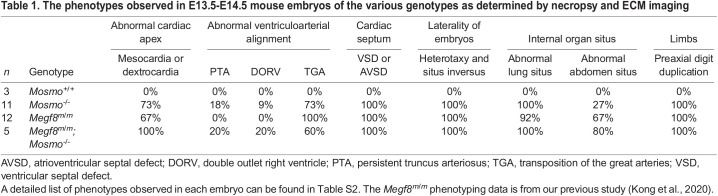
The phenotypes observed in E13.5-E14.5 mouse embryos of the various genotypes as determined by necropsy and ECM imaging

**Fig. 3.**
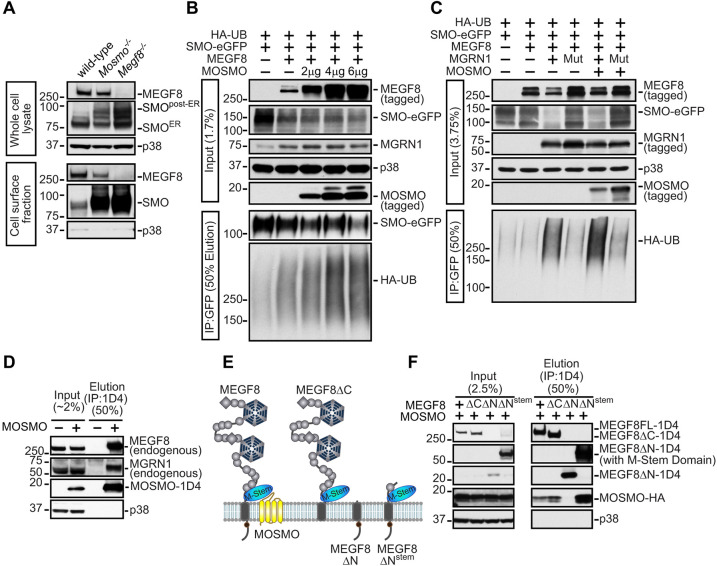
**MOSMO interacts with MEGF8 and regulates its abundance at the cell surface.** (A) Abundance of SMO and MEGF8 protein in whole-cell lysates (top, 6.25% input) and on the cell surface [bottom, cell surface biotinylation and streptavidin immunoprecipitation (IP), 50% elution] in NIH/3T3 cells of the indicated genotypes. The cytoplasmic protein p38 serves as a loading control. (B,C) SMO ubiquitylation was assessed after transient co-expression of the indicated proteins in HEK293T cells, as described in our previous study (Kong et al., 2020). Cells were lysed under denaturing conditions, SMO was purified by immunoprecipitation, and the amount of HA-tagged ubiquitin (HA-UB) covalently conjugated to SMO was assessed using immunoblotting with an anti-HA antibody. In B, assays were carried out in the presence of endogenous MGRN1 and increasing amounts of transfected MOSMO. Assays in D co-transfected either wild-type MGRN1 or catalytically inactive MGRN1 (MGRN1^Mut^) to show that SMO ubiquitylation was dependent on the function of MGRN1. (D) Endogenous MEGF8 and MGRN1 co-purified with 1D4-tagged MOSMO immunoprecipitated from *Mosmo^−/−^* NIH/3T3 cells stably expressing MOSMO-1D4 (Fig. S4A,B). (E) Domain graphics depict the simplified modular architecture of wild-type MEGF8 (leftmost image) and its engineered variants (right three images). Gray circles denote the linearly connected EGFL and PSI repeats, with interspersed six-bladed β-propeller domains (hexagons) and CUB domains (diamonds), while the juxtamembrane M-Stem domain is a blue oval. The larger globular structures of the β-propeller, CUB and M-Stem folds all have closely spaced N- and C-termini, so they appear as pendant-like inserts into the long EGFL and PSI chain. A proposed mode of interaction between the M-Stem domain of MEGF8 and the extracellular domain of MOSMO is depicted (leftmost), based on modeling described in Fig. S5. (F) A series of truncation mutants of MEGF8 (shown in F) were used to identify the MEGF8 domain that binds to MOSMO. The interaction between HA-tagged MOSMO and these 1D4-tagged MEGF8 variants was assessed by transient expression in HEK293T cells followed by an anti-1D4 IP and immunoblotting to measure the amount of co-precipitated MOSMO-HA (right).

A clue to the biochemical function of MOSMO came from the observation that the abundance of cell-surface MEGF8 was reduced in *Mosmo^−/−^* cells. Cell-surface biotinylation analysis demonstrated that the loss of MOSMO reduced MEGF8 (and concomitantly increased SMO) at the plasma membrane; Fig. 3A). These results are consistent with the model that MOSMO promotes ubiquitylation of SMO via the MEGF8-MGRN1 complex by increasing MEGF8 levels at the cell surface. Indeed, co-expression of MOSMO increased the ubiquitylation of SMO by the MEGF8-MGRN1 complex in an assay reconstituted in HEK293T cells (Fig. 3B,C). The influence of MOSMO on MEGF8 activity and localization led us to test the possibility of a physical interaction between the two proteins. Epitope-tagged MOSMO stably expressed in *Mosmo^−/−^* cells (Fig. S4A,B) was co-immunoprecipitated with both endogenous MEGF8 and MGRN1 (Fig. 3D).

The interaction between MEGF8 and MOSMO mapped to the previously unrecognized β-strand-rich MEGF8-Stem (hereafter ‘M-Stem’) domain of MEGF8, positioned at the extracellular end of the transmembrane helix (Fig. 3E,F). Using sensitive deep-learning-based structure prediction methods (Yang et al., 2020), the M-Stem domain is predicted to adopt a novel β-jellyroll topology, but is not closely related to any previously described domain with such a structure (Fig. S5A,B). We propose that the M-Stem domain of MEGF8 docks to the compact extracellular β-sheet surface of MOSMO. As MOSMO is distantly related to the claudins (Pusapati et al., 2018), we modeled this interaction based on the binding of the Clenterotox domain of *Clostridium perfringens* enterotoxins (which adopt a fold related to CUB domains) to claudin 3, claudin 4 and claudin 19 (Suzuki et al., 2017) (Fig. S5C, left). Taken together, we propose that MOSMO, MEGF8 and MGRN1 together form a membrane-tethered E3 ligase complex (hereafter the ‘MMM complex’, Fig. S5C, right) that modulates the strength of Hh signaling by regulating levels of SMO at the cell surface and primary cilium.

### *Mosmo^−/−^* limb phenotypes can be suppressed by the small-molecule SMO inhibitor vismodegib

**Fig. 4.**
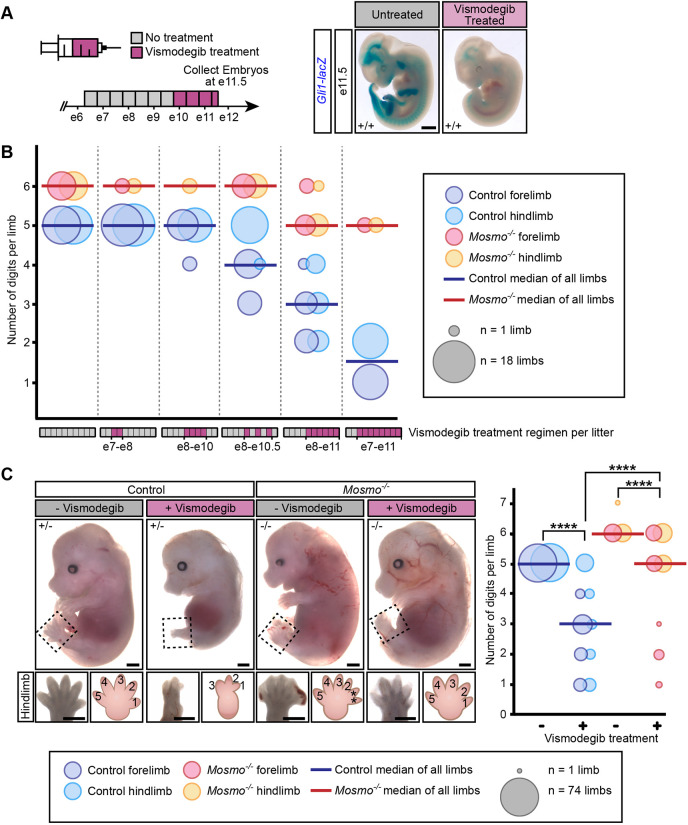
**Reducing Hh signaling strength with the SMO rescues limb defects in *Mosmo^−/−^* embryos.** (A) Vismodegib administered every 12 h for ∼2 days from E9.75 to E11.5 (diagrammed on the left, with purple used to shade developmental windows for drug exposure) reduced *Gli1-lacZ* expression in wild-type embryos (right). Scale bar: 1 mm. (B) Numbers of digits per limb of E14.5 embryos from six individual litters exposed to increasing amounts of vismodegib (represented by purple shading in the developmental time courses depicted on the *x*-axis). The area of each bubble is proportional to the number of limbs included in the analysis. Bold horizontal lines represent the median number of digits per limb in control (*Mosmo^+/+^* and *Mosmo^+/−^*, blue) and *Mosmo^−/−^* (red) embryos. (C) Representative images (left) of E14.5 control and *Mosmo^−/−^* embryos treated with or without vismodegib every 12 h for 3 (E8.25-E11.25) or 4 (E7.25-E11.25) days. Scale bars: 1 mm. Bubble plot (right) used to depict the numbers of digits per limb of control (*Mosmo^+/+^* and *Mosmo^+/−^*, blue) and *Mosmo^−/−^* (red) embryos treated with vismodegib. Statistical significance was determined using the Kruskal–Wallis test (****P<0.0001).

As a component of the MMM complex, MOSMO attenuates Hh signaling activity in the developing embryo (Fig. 2A-C) by clearing SMO from the cell surface and primary cilium (Fig. 2D,E). However, MOSMO may also regulate other cellular pathways and processes. Thus, we sought to investigate whether the developmental defects (i.e. polydactyly and CHDs) observed in *Mosmo^−/−^* embryos (Figs 1 and 3A) were caused by elevated Hh signaling. We took the unconventional approach of using an FDA-approved small-molecule SMO antagonist (vismodegib) to reduce Hh signaling strength at crucial periods of embryonic development. There are many advantages to using small-molecule inhibitors in an embryonic system. First, previous studies have shown that the SMO inhibitors cyclopamine and vismodegib are potent placenta-permeable teratogens that can induce embryonic defects in Hh-dependent tissues when delivered orally to the pregnant mother (Binns et al., 1963; Lipinski et al., 2008). Second, a small-molecule strategy allows us to selectively reduce Hh signaling during defined developmental periods to target events like limb digitation and heart looping. Last, by changing the treatment dose and frequency, a small-molecule inhibitor allows us to experimentally adjust Hh signaling activity as needed, based on the phenotypic outcomes (Heyne et al., 2015). In a proof-of-concept experiment, we found that treatment of pregnant mice with vismodegib for about 2 days (e9.75-e11.5) reduced *Gli1-lacZ* expression in wild-type embryos when compared with untreated controls (Fig. 4A).

*Shh* is transiently expressed along the posterior margin from ∼E9.5-E12 in the murine forelimb and ∼E10-E12.5 in the hindlimb (Büscher et al., 1997; Zhu et al., 2008). Hh signaling plays an established role in the anterior-posterior patterning of digits. Oligodactyly (digit loss) can be caused by exposure to a Hh antagonist or to loss of *Shh* expression during a crucial period of limb development (Heyne et al., 2015; Zhu et al., 2008). Conversely, preaxial polydactyly can arise from elevated Hh signaling activity caused by an increase in *Shh* or reduction in *Gli3* expression (Hill and Lettice, 2013; Hui and Joyner, 1993). To determine whether the fully penetrant preaxial polydactyly observed in the *Mosmo^−/−^* embryos was due to elevated Hh signaling activity, pregnant dams from *Mosmo^+/−^*×*Mosmo^+/−^* crosses were exposed to vismodegib for varying durations of time and E14.5 embryos were collected to examine digit patterning. There were no defects in limb patterning in embryos that received no treatment or embryos that were treated with vismodegib before E9.5 (prior to *Shh* limb expression). However, when embryos were treated with vismodegib after E9.5, increasing the duration of drug treatment resulted in progressively greater oligodactyly (Fig. 4B). The exquisitely graded nature of Hh signaling was vividly demonstrated by the striking dose-response relationship between vismodegib exposure and digit number. Interestingly, vismodegib treatment had a profoundly different impact on limb patterning, even between embryos in the same litter (Fig. 4B). Although vismodegib caused severe oligodactyly in control (*Mosmo^+/+^* and *Mosmo^+/−^*) embryos, the same dose often corrected the polydactyly in *Mosmo^−/−^* embryos, resulting in embryos with normal limbs bearing five digits (Fig. 4B,C). We conclude that the polydactyly observed in *Mosmo^−/−^* embryos is indeed due to an elevation of SMO activity as it can be reversed by a direct SMO antagonist (Fig. S6A).

### *Mosmo^−/−^* cardiac phenotypes can be partially suppressed by SMO inhibitors

**Fig. 5.**
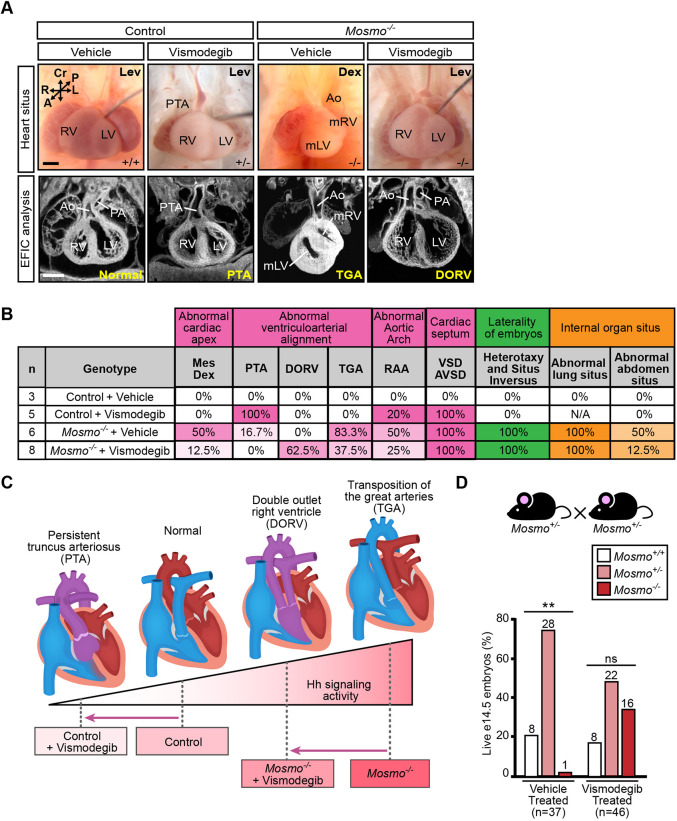
**Reducing Hh signaling strength partially rescues heart defects in *Mosmo^−/−^* embryos.** (A) Representative necropsy (top row) and ECM (bottom row) images of embryonic hearts from E14.5 control (*Mosmo^+/+^* and *Mosmo^+/−^*) and *Mosmo^−/−^* embryos treated with vismodegib or a vehicle control. Vismodegib was administered every 12 h for 3 (E8.25-E11.25) or 4 (E7.25-E11.25) days. Scale bars: 200 µm. (B) Summary of heart malformations and left-right patterning defects in E14.5 control (*Mosmo^+/+^* and *Mosmo^+/−^*) and *Mosmo^−/−^* mouse embryos treated with either vehicle or vismodegib every 12 h for 3 (E8.25-E11.25) or 4 (E7.25-E11.25) days. Lung situs could not be determined (N/A) in vismodegib-treated control embryos due to cystic and hypoplastic lungs. Ao, aorta; DORV, double outlet right ventricle; LV, left ventricle; mLV, morphological left ventricle; mRV, morphological right ventricle; PA, pulmonary artery; PTA, persistent truncus arteriosus; RV, right ventricle; TGA, transposition of the great arteries. A detailed list of phenotypes observed in each embryo can be found in Table S4. (C) A proposed model for how a combination of genotype and SMO inhibition by vismodegib influences Hh signaling strength and consequently ventriculoarterial alignment in developing embryos. (D) Viability of E14.5 embryos from *Mosmo^+/−^*×*Mosmo^+/−^* crosses treated with vehicle or vismodegib every 12 h for 3 (E8.25-E11.25) or 4 (E7.25-E11.25) days. Statistical significance was determined using the chi-squared test (ns>0.05, **P<0.01) and *n*=number of live embryos collected. See Table S5 for full details.

The ability of vismodegib to rescue the polydactyly phenotype led us to test its effects on the complex CHD phenotypes contributing to the lethality of *Mosmo^−/−^* embryos (Fig. 1A,F). Conotruncal heart defects are a group of malformations that arise due to defects in outflow tract development. All of the *Mosmo^−/−^* embryos had conotruncal heart defects, most commonly transposition of the great arteries (TGAs) (Table 1A and Table S2). Hh signaling plays a crucial role in multiple aspects of outflow tract development, including: (1) maintenance of cardiac progenitor proliferation and identity within the secondary heart field (which contributes to the outflow tract) (Dyer and Kirby, 2009; Rowton et al., 2020 preprint); (2) survival of migratory cardiac neural crest cells (Washington Smoak et al., 2005); and (3) proper septation of the outflow tract (Goddeeris et al., 2007). To determine whether the conotruncal heart defects observed in the *Mosmo^−/−^* embryos are due to elevated Hh signaling, we first needed to identify the critical time window during gestation when outflow tract development is sensitive to vismodegib. Loss-of-function mutations in *Shh* result in a failure of the primitive truncus to properly divide into the aorta and pulmonary artery (persistent truncus arteriosus, PTA) (Washington Smoak et al., 2005). Building on this information, we found that vismodegib administered from E7.25/E8.25 to E11.25 caused PTA in all control (*Mosmo^+/+^* and *Mosmo^+/−^*) embryos (Fig. 5A,B). In contrast, *Mosmo^−/−^* embryos exposed *in utero* to the same vismodegib regimen did not develop PTA, suggesting that these mutant embryos were resistant to the SMO antagonist because of elevated SMO abundance (Fig. 5B).

Indeed, vismodegib administration actually improved the CHD phenotypes characteristically seen in *Mosmo^−/−^* embryos. Instead of the predominant TGA phenotype seen in untreated *Mosmo^−/−^* embryos, SMO inhibition shifted the phenotype to DORV, an overlapping conotruncal malformation also associated with defective ventriculoarterial alignment but of reduced severity compared with TGA (Fig. 5A-C and Table S4). Vismodegib treatment in *Mosmo^−/−^* embryos also had a corrective effect on the position of the heart, reducing the incidence of mesocardia, dextrocardia and right aortic arch (RAA). Notably, these defects are all reflective of improper left-right cardiac morphogenesis (Fig. 5A,B and Table S4). Perhaps the most compelling evidence that vismodegib treatment improved *Mosmo^−/−^* heart development and function came from the analysis of embryo survival. The number of *Mosmo*^−/−^ embryos that survived to E14.5 was 16-fold higher in vismodegib-treated litters compared with vehicle-treated litters (Fig. 5D and Table S5). We speculate that the incomplete rescue of CHDs may be due to the challenges of delivering the correct dose of vismodegib at the correct time to impact the multiple (temporally different) points in development when Hh signaling is required for heart development.

### Neural patterning in *Mosmo^−/−^* embryos is resistant to SMO inhibitors

The loss of MOSMO did not cause defects in all tissues that are known to require SHH for their patterning. Dorsal-ventral patterning of the developing spinal cord is coordinated by a gradient of SHH that is secreted initially from the notochord and later from the floor plate. A large body of literature has shown that neural progenitors adopt different cell fates depending on the magnitude of their exposure to SHH. Increasing the concentration of SHH or the duration of SHH exposure results in an expansion of ventral neural cell fates (Dessaud et al., 2007). Unexpectedly, staining with a panel of cell-type specific markers revealed normal neural tube patterning in E8.5 and E10.5 *Mosmo*^−/−^ embryos (Fig. S6B and data not shown). The integrity of neural patterning was maintained despite the fact that the loss of MOSMO resulted in markedly elevated levels of ciliary SMO along the entire dorsal-ventral axis of the developing spinal cord (Fig. S6C). In wild-type embryos, SMO accumulates in the cilia of only the ventral-most progenitor cells (floor plate and p3 progenitors) of the developing spinal cord, cells that are exposed to the highest concentrations of SHH (Kong et al., 2015). However, ciliary SMO levels were elevated in all progenitor domains in *Mosmo*^−/−^ embryos, even those distant from the SHH source at the floor plate (Fig. S6C).

**Fig. 6.**
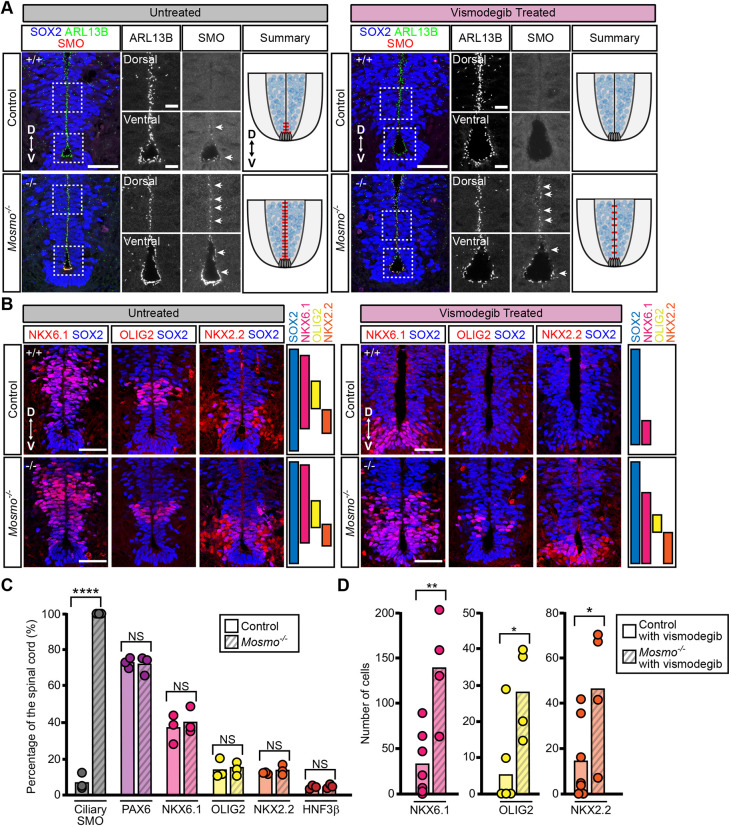
**The developing spinal cords of *Mosmo^−/−^* embryos are resistant to SMO inhibition.** Neural tube patterning was assessed using confocal fluorescence microscopy to image markers that define neural progenitor populations in sections of the ventral spinal cord from E11.5 control (*Mosmo^+/+^* and *Mosmo^+/−^*) and *Mosmo^−/−^* embryos treated with or without vismodegib. (A) Immunofluorescence (IF) was used to evaluate SMO abundance (red) in primary cilia (ARL13B, green) within neural progenitors (SOX2, blue) of untreated E11.5 embryos (left) and vismodegib-treated embryos (right). White arrows indicate regions where SMO is seen in cilia; red lines indicate the regions of the developing neural tube where ciliary SMO is observed. Scale bars: 100 µm in merged panels; 50 µm in zoomed displays. (B) IF was used to assess the abundance and distribution of NKX6.1^+^, OLIG2^+^ and NKX2.2^+^ (red) neural progenitor cells (SOX2, blue) of untreated E11.5 embryos (left) and vismodegib-treated embryos (right). Images represent three serial sections taken from a single representative embryo. Scale bars: 50 µm. (C) Summary of the dorsal-ventral distribution of ciliary SMO, PAX6, NKX6.1, OLIG2, NKX2.2 and HNF3β from untreated E10.5 control (*Mosmo^+/+^* and *Mosmo^+/−^*) (*n*=3) and *Mosmo^−/−^* (*n*=3) embryos. See Fig. S6B,C for representative images. (D) Quantification of NKX6.1^+^, OLIG2^+^ and NKX2.2^+^ spinal neural progenitors from vismodegib-treated E11.5 control (*n*=7) and *Mosmo^−/−^* (*n*=4) embryos. (C,D) Each point represents one embryo. Data are the mean and the statistical analysis between the two groups was determined using an unpaired two-tailed *t*-test (ns>0.05, *P<0.05, **P<0.01 and ****P<0.0001).

While the patterning of ventral spinal progenitors was indistinguishable in control (*Mosmo^+/+^* and *Mosmo^+/−^*) and *Mosmo*^−/−^ embryos, a dramatic difference was uncovered when these embryos were exposed to vismodegib. Similar to previous experiments, pregnant dams from *Mosmo^+/−^*×*Mosmo^+/−^* crosses were exposed to vismodegib from E7.75 to E11.25 and then collected at E11.5 for spinal cord analysis. In control (*Mosmo^+/+^* and *Mosmo^+/−^*) embryos, vismodegib caused the loss of ciliary SMO (Fig. 6A) and a profound reduction of the ventral-most progenitor domains (OLIG2^+^ and NKX2.2^+^ neural progenitors) that require SHH for their specification (Fig. 6B). These same ventral cell types are also lost in *Smo*^−/−^ and *Shh*^−/−^ embryos, confirming that vismodegib mimics the genetic disruption of Hh signaling (Chiang et al., 1996; Wijgerde et al., 2002). In contrast, *Mosmo*^−/−^ embryos from the same litter (exposed to the same vismodegib regimen), maintained OLIG2^+^ and NKX2.2^+^ progenitors (Fig. 6B-D). Although the OLIG2^+^ and NKX2.2^+^ progenitors were present, they were shifted to more ventral positions within the spinal cord in vismodegib-treated *Mosmo^−/−^* embryos, consistent with partial SMO inhibition. Thus, the loss of a single gene, *Mosmo*, can influence the impact of an established teratogen (vismodegib) on neural tube patterning, likely by increasing the abundance of SMO, the protein target of the teratogen.

## DISCUSSION

**Fig. 7.**
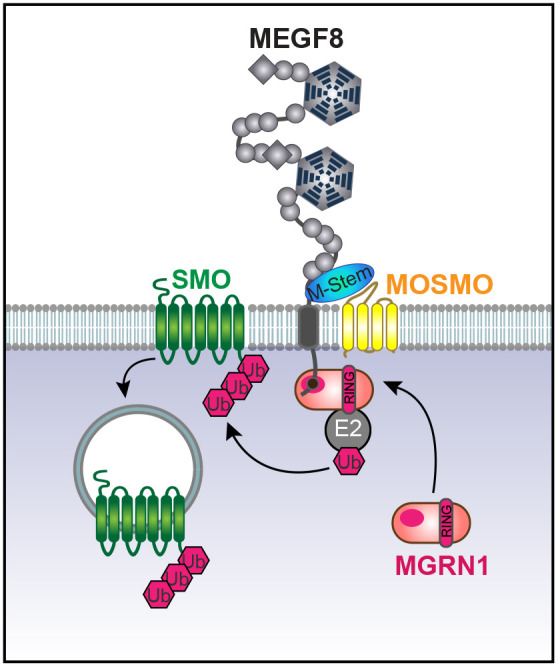
**The regulation of cell surface Smoothened (SMO) by the MMM complex.** MOSMO binds to the M-Stem domain of MEGF8. Together, MOSMO, MEGF8 and MGRN1 form a membrane-tethered E3 ligase complex (the MMM complex) that regulates the sensitivity of a target cell to Hh ligands by regulating levels of SMO at the cell surface and primary cilium.

As MOSMO was an uncharacterized protein, we ablated *Mosmo* in the mouse and discovered that it is essential for embryonic development. In the absence of MOSMO, SMO is enriched in the primary cilia of all tissues, rendering cells hypersensitive to endogenous SHH. Consequently, *Mosmo^−/−^* embryos suffer from severe developmental defects, including heterotaxy, skeletal abnormalities and congenital heart defects (CHDs). The MMM complex composed of MOSMO, MEGF8 and MGRN1 anchors a signaling pathway that regulates the sensitivity of target cells to Hh morphogens (Fig. 7). All three components of the MMM complex were originally identified as attenuators of Hh signaling in our genome-wide CRISPR screens (Pusapati et al., 2018). The phenotypic similarities between *Mosmo^−/−^*, *Megf8^−/−^* and *Mgrn1^−/−^*;*Rnf157^−/−^* cells and mouse embryos supports the model that the MMM proteins function in the same pathway. We previously found that MGRN1 interacts with MEGF8, forming a membrane-tethered ubiquitylation complex that targets SMO for degradation (Kong et al., 2020). Here, we report that MOSMO interacts with MEGF8 to facilitate its accumulation at the cell surface. In general terms, this action of Mosmo might be compared with that of other members of the claudin-like superfamily of 4-pass transmembrane (4TM) proteins to which it belongs. These include the calcium channel γ subunits that play a role in the localization of transmembrane calcium-channel AMPA receptors to the synapse (Chen et al., 2007). More specifically, another related protein, LHFPL4, forms a ternary complex with ionotropic GABA_A_ receptors and neuroligin 2 (NL2) and helps localize the former receptor to synaptic membranes (Yamasaki et al., 2017). Similarly, another related protein, LHFPL5, forms a complex with the transmembrane channel-like protein isoform 1 (TMC1) in the auditory stereocilia and might play a role in its localization to that structure (Beurg et al., 2015; Yu et al., 2020). Thus, beyond their roles in tight junctions, members of the claudin-like superfamily, including MOSMO, might play a general role in the accumulation of specific complexes at the membrane.

### The MMM complex shapes the Hh signaling gradient

*Mosmo* is widely expressed in the mouse embryo (Fig. S1) and MOSMO deficiency results in a dramatic increase in ciliary SMO in all tissues we examined (Fig. 2D,E). However, *Mosmo^−/−^* embryos did not show indiscriminate Hh signaling activation in all tissues. The pattern of elevation in Hh signaling activity due to loss of *Mosmo* is very different from the pattern seen in embryos lacking the well-studied Hh negative regulators PTCH1 and SUFU (Cooper et al., 2005; Goodrich et al., 1997; Svärd et al., 2006). Although Hh signaling is fully activated in most tissues in *Ptch1^−/−^* and *Sufu^−/−^* embryos, Hh signaling is amplified selectively in SHH-exposed tissues in *Mosmo^−/−^* embryos. We propose that the purpose of the MMM complex is to attenuate the gradient of Hh signaling strength in tissues, rather than to suppress basal signaling activity.

The loss of *Mosmo* clearly has tissue-specific effects. Although Hh signaling is required for the patterning of many tissues, development of the limbs, heart and skeleton was more severely affected by this elevation in pathway activity than the ventral spinal cord. These differences may reflect whether patterning in a tissue depends on transcriptional de-repression or activation. The patterning of the limb bud is driven by the de-repression of downstream target genes due to a loss of GLI transcriptional repressors (GLIR) (Litingtung et al., 2002; te Welscher et al., 2002). In contrast, the patterning of the ventral spinal cord is primarily driven by the activation of downstream target genes by GLI transcriptional activators (GLIA) (Stamataki et al., 2005). We speculate that loss of the MMM complex potentiates Hh signaling by reducing GLIR levels, rather than by elevating GLIA (Kong et al., 2019; Niewiadomski et al., 2014). In support of this notion, *Mosmo^−/−^* mouse embryos have some of the same phenotypes as *Gli3^−/−^* embryos. GLI3 is proteolytically processed to generate GLI3R, the predominant transcriptional repressor in Hh signaling. As seen in *Mosmo^−/−^* embryos, a loss of GLI3 results in polydactyly (Hui and Joyner, 1993; Johnson, 1967), but no changes in the patterning of the ventral spinal cord (Persson et al., 2002). Overall, our results suggest that limb, heart and skeleton development are particularly susceptible to subtle changes in Hh signaling strength caused by either genetic perturbations or environmental exposures to teratogens such as vismodegib. This heightened sensitivity could underlie the sporadic nature of CHDs and contribute to its variable penetrance and expressivity.

### The role of the MMM complex in left-right patterning

Left-right patterning defects, which manifest as heterotaxy with randomization of visceral organ situs, are frequently associated with severe CHDs in humans, suggesting that the signals that specify the left/right body axis also play a role in regulating heart development (Li et al., 2015; Lin et al., 2014). Left-right patterning defects and complex heart malformations are prominent phenotypes common to all the MMM mutant mouse lines (Cota et al., 2006; Zhang et al., 2009). Although vismodegib treatment was able to fully rescue the *Mosmo^−/−^* polydactyly phenotype (Fig. 4), demonstrating that digit duplication is a product of elevated Hh signaling, it failed to fully rescue heterotaxy phenotypes (Fig. 5B). With regard to the heart, vismodegib partially rescued cardiac *situs* and improved outflow tract malalignment defects, with DORV seen instead of TGA. This finding is intriguing as there is an ongoing debate about whether DORV and TGA are related phenotypes arising from a disturbance in left-right patterning. Our findings suggest that these two phenotypes are indeed developmentally related and may be influenced by changes in Hh signaling strength.

Our failure to rescue heterotaxy phenotypes with vismodegib could be because (1) the MMM complex regulates receptors involved in other signaling pathways or (2) we did not deliver vismodegib during the correct time window in development. Interestingly, a study recently found that the conditional deletion of *Megf8* in all known cardiac cell lineages did not reproduce the heart defects observed in the global *Megf8* knockout (Wang et al., 2020). These data suggest that Megf8 is required for cardiac development at a time point earlier than cardiac organogenesis and supports the possibility that the heart defects seen in MMM mutant mice are a consequence of disrupted left-right patterning earlier in development. Further studies are needed to identify both the cell types and the critical time periods that are relevant to the function of the MMM complex during development of various tissues.

### Mutations and teratogens converge on Hh signaling to determine the penetrance of birth defects

A central principle of teratology holds that susceptibility to birth defects depends on interactions between the genotype of the embryo and environmental exposures (Finnell, 1999). However, the molecular mechanisms that underlie these gene-teratogen interactions remain largely unknown. Our work on the MMM complex provides one molecular mechanism that explains how mutations and small molecules can interact to influence birth defect outcomes by their combined effects on Hh signaling. Hh ligands function as classical morphogens that direct developmental processes in a manner dependent on the strength of signal in target cells. This simple and elegant mechanism for regulating the patterning and morphogenesis of target tissues, however, also leaves the development of these tissues vulnerable to even small shifts in signaling strength.

Mutations in MMM complex genes cause elevated sensitivity of cells to Hh ligands by increasing the abundance of SMO on the cell surface. Even between embryos in the same litter, *Mosmo* mutations have a profound influence on the teratogenicity of vismodegib, a direct small molecule SMO antagonist. Vismodegib can cause a variety of structural birth defects: neural tube patterning errors, oligodactyly and cardiac outflow tract abnormalities such as PTA (Figs 4C and 5B). Remarkably, the developing neural tube and cardiac outflow tracts of *Mosmo^−/−^* embryos are resistant to vismodegib exposure compared with wild-type embryos (Figs 5 and 6), likely because the elevated SMO abundance protects these embryos from its teratogenic effects. In the limb and the heart, vismodegib has an even more striking effect on *Mosmo^−/−^* embryos: it rescues structural birth defect phenotypes and improves overall embryo survival, likely by reducing SMO activity to levels that allow normal development. We propose that total SMO activity in target cells, which is influenced by both SMO protein abundance and exposure to a SMO antagonist, determines birth defect outcomes. Elevated SMO abundance in MMM-mutant embryos can be overcome by reducing SMO activity with vismodegib. Conversely, the reduction in SMO activity caused by vismodegib can be overcome by increasing SMO protein abundance. Thus, gene-environment interactions can arise when genetic factors change the abundance of the protein target of a teratogen.

Previous studies have provided evidence that mutations and environmental exposures can influence development of the face and brain by their combined effects on Hh signaling. Ethanol and the pesticide component piperonyl butoxide (PBO) are exogenous agents that suppress Hh signaling. Early embryonic exposure to high concentrations of ethanol and PBO can cause craniofacial abnormalities and holoprosencephaly (an incomplete division of the forebrain, HPE): phenotypes associated with reduced Hh signaling activity (Ahlgren et al., 2002; Everson et al., 2019; Wang et al., 2012). Embryos exposed to low concentrations of ethanol and PBO develop normally. However, even at low concentrations the teratogenic effects of these agents begin to emerge when they are administered to mice carrying mutations in Hh pathway components (*Shh^+/−^*, *Gli2^+/−^* or *Cdon^−/−^* embryos) (Everson et al., 2019; Hong and Krauss, 2017; Kietzman et al., 2014).

In summary, the graded dose-dependent influence of Hh signaling on developmental patterning and morphogenesis explains how gene-teratogen interactions can conspire to modulate the penetrance and expressivity of birth defects by tuning the strength of Hh signaling. A provocative corollary that follows from this idea is that it may be possible to rescue structural birth defect phenotypes by using small molecules (e.g. SMO agonists or antagonists) to re-calibrate Hh signaling strength to the optimal levels required to support normal development. As these drugs are teratogens themselves, they would need to be delivered during defined time periods in development, at precise doses and to embryos of defined genotypes.

## MATERIALS AND METHODS

### NIH/3T3 and HEK293T cell culture

Flp-In-3T3 (referred to as ‘NIH/3T3’ cells throughout the text) and HEK293T cell lines were purchased from Thermo Fisher Scientific and the American Type Culture Collection (ATCC), respectively. Information on the gender of the cell lines is not available. As previously described (Kong et al., 2020), NIH/3T3 and HEK293T cells were cultured in Complete Medium: Dulbecco's Modified Eagle Medium (DMEM) containing high glucose (Thermo Fisher Scientific, Gibco) supplemented with 10% fetal bovine serum (FBS) (MilliporeSigma), 2 mM L-glutamine (Gemini Bio-Products), 1 mM sodium pyruvate (Thermo Fisher Scientific, Gibco), 1× MEM non-essential amino acids solution (Thermo Fisher Scientific, Gibco), and penicillin (40 U/ml) and streptomycin (40 µg/ml) (Gemini Bio-Products). The NIH/3T3 and HEK293T cells were rinsed once with sterile PBS and then passaged using 0.05% Trypsin/EDTA (Gemini Bio-Products). Cells were housed at 37°C in a humidified atmosphere containing 5% CO_2_. Cell lines and derivatives were free of mycoplasma contamination, as determined by PCR using the Universal Mycoplasma Detection Kit (ATCC).

### Generation of primary mouse embryonic fibroblasts

Primary mouse embryonic fibroblasts (pMEFs) were generated using a modified published protocol (Durkin et al., 2013). Briefly, E13.5 embryos were harvested from *Mosmo^+/−^*×*Mosmo^+/−^* crosses and rinsed thoroughly with sterile PBS. Using forceps, the head and internal organs were removed. The embryos were then separated into individual dishes and the tissue was physically minced into small bits in 0.25% Trypsin/EDTA (Thermo Fisher Scientific, Gibco) using a sterile razor blade. Using initially a 5 ml serological pipette and later a P1000 pipette tip, the minced tissue was pipetted up and down several times to further break up the tissue, and the dishes were placed in a 37°C tissue culture incubator for 10-15 min. If there were still large tissue pieces present, the minced tissue was pipetted further and the dish was placed in the incubator for an additional 5-10 min. The trypsin was then deactivated using Complete Medium (containing 10% FBS). The cells were then centrifuged, resuspended in fresh Complete Medium and plated. Each clonal cell line represents pMEFs generated from a single embryo. Analysis of *Mosmo^−/−^* pMEFs was always performed with pMEFs prepared from *Mosmo^+/+^* and *Mosmo^+/−^* littermate controls. The gender of the embryos were not determined. Cells were housed at 37°C in a humidified atmosphere containing 5% CO_2_.

### Hh signaling assays in NIH/3T3 cells and primary fibroblasts

Hh signaling assays were performed as previously described (Kong et al., 2020). Briefly, NIH/3T3 cells and pMEFs were first grown to confluence in Complete Medium (containing 10% FBS) and then ciliated by changing to Low Serum Medium (Complete Medium containing 0.5% FBS) overnight. NIH/3T3 cells were treated with either no SHH, a low concentration of SHH (1 nM) or a high concentration of SHH (25 nM) prepared in Low Serum Medium. SHH treatment durations varied based on application: 12 h prior to fixation for NIH/3T3 immunofluorescence assays, 24 h prior to lysis for NIH/3T3 western blot assays or NIH/3T3 RNA extraction, and 48 h prior to pMEF experimentation (immunofluorescence, western blot and RNA extraction).

Hh signaling activity was measured using real-time quantitative reverse transcription PCR (qRT-PCR). RNA was extracted from NIH/3T3 cells and pMEFs using TRIzol reagent (Thermo Fisher Scientific, Invitrogen) as previously described (Rio et al., 2010). Equal amounts of RNA were used as a template for cDNA synthesis using the iScript Reverse Transcription Supermix (Bio-Rad Laboratories). qRT-PCR for mouse *Gli1* and mouse *Gapdh* was performed on a QuantStudio 5 Real-Time PCR System (Thermo Fisher Scientific) with the following custom designed primers: mouse *Gli1* (fwd, 5′-CCAAGCCAACTTTATGTCAGGG-3′; rev, 5′-AGCCCGCTTCTTTGTTAATTTGA-3′) and mouse *Gapdh* (fwd, 5′-AGTGGCAAAGTGGAGATT-3′; rev, 5′-GTGGAGTCATACTGGAACA-3′). For all qRT-PCR experiments, *Gli1* transcript levels were calculated relative to *Gapdh* and reported as a fold change across conditions using the comparative C_T_ method (ΔΔC_T_ method).

### Generation of knockout cell lines

Clonal *Mosmo^−/−^*, *Megf8^−/−^*, *Mgrn1^−/−^* and *Mgrn1^−/−^*;*Rnf157^−/−^*NIH/3T3 lines were previously generated and validated (Kong et al., 2020; Pusapati et al., 2018). Clonal double knockout *Mosmo^−/−^*;*Megf8^−/−^* NIH/3T3 cell lines were generated using the dual sgRNA strategy to target *Megf8* in *Mosmo^−/−^* NIH/3T3 cells as previously described (Pusapati et al., 2018). Briefly, sgRNAs targeting *Megf8* (5′-TGCCTTCTCTGCCCGAATTG-3′ and 5′-ATAACTTCTCCACGAACACC-3′) were cloned into pSpCas9(BB)-2A-GFP (Addgene) (Ran et al., 2013) and pSpCas9(BB)-2A-mCherry, and transfected into NIH/3T3 cells using X-tremeGENE 9 DNA transfection reagent (Roche Molecular Systems). Five days post transfection, GFP and mCherry double-positive single cells were sorted into a 96-well plate using a FACSAria II at the Stanford Shared FACS Facility. To detect the GFP, a 488 nm (blue) laser was used with a 530/30 filter and B530 detector. To detect the mCherry, a 561 nm (yellow) laser was used with a 616/23 filter and G616 detector. Clonal lines were screened by PCR (forward primer, 5′-CCTCATGCTTGTCCCTTGTT-3′; reverse primer, 5′-GGAGTGTGGGCAAGAAGAAG-3′) to detect excision of the genomic DNA (196 bp) between the two sgRNA cut sites. Knockout of MEGF8 was further confirmed by immunoblotting (Fig. S4D).

### Generation of stable cell lines expressing transgenes

*Mosmo^−/−^* NIH/3T3 cells with stable addback of tagged MOSMO (featured in Fig. S4A,B) were generated using the lentiviral expression system as previously described (Kong et al., 2020). Briefly, to generate lentivirus, four million HEK293T cells were seeded onto a 10 cm plate and transfected 24 h later with 1 µg pMD2.G (Addgene), 5 µg psPAX2 (Addgene) and 6 µg of the *Mosmo-1D4* pLenti CMV Puro DEST construct using 36 µl of 1 mg/ml polyethylenimine (PEI) (Polysciences). Approximately 48 h post transfection, the lentivirus was harvested and filtered through a 0.45 µm filter. 2 ml of the filtered lentivirus solution was mixed with 2 ml of Complete Medium containing 16 µg/ml polybrene (MilliporeSigma). The diluted virus was then added to NIH/3T3 cells seeded on 6-well plates. Approximately 48 h post infection, cells were split and selected with puromycin (2 µg/ml) for 5-7 days or until all the cells on the control plate were dead.

### Established mouse lines

All mouse studies were conducted using animal study protocols approved by the Institutional Animal Care and Use Committee (IACUC) of Stanford University, the University of Pittsburgh and the McLaughlin Research Institute for Biomedical Sciences. *Gli1^tm2Alj^* mice (referred to in the paper as *Gli1^lacz^*) (MGI:2449767) and *Megf8^C193R/C193R^* mice (referred to in the paper as *Megf8^m/m^*) (MGI:3722325) have been described previously (Bai et al., 2002; Zhang et al., 2009). *Gli1^lacz^* mice were genotyped using the following primers: Fwd (common), 5′-GGGATCTGTGCCTGAAACTG-3′; Rev (wild type), 5′-AGGTGAGACGACTGCCAAGT-3′; and Rev (mutant), 5′-TCTGCCAGTTTGAGGGGACGAC-3′.

### Generation and genotyping of *Mosmo^−/−^* mutant mice

*Mosmo^+/−^* mice were generated by the Stanford Transgenic Knockout and Tumor Model Center using CRISPR/Cas9-mediated genome editing. In brief, four gRNAs were designed to delete exon 1 of *Mosmo* to create a downstream reading frame shift in exon 2 and exon 3, and ultimately remove its function (Fig. S2A). The four guide RNAs (5′-CCGGCGCGCGGTTTCGCTTC-3′, 5′-CGCGGTTTCGCTTCCGGGTG-3′, 5′-CCCCGGGTCGGCGATCCCGA-3′ and 5′-CCCTCGGGATCGCCGACCCG-3′) and CAS9 protein were obtained from Integrated DNA Technologies. A ribonucleoprotein (RNP) injection mix was prepared, consisting of gRNAs (15 ng/μl) and CAS9 protein (30 ng/μl), and introduced into C57BL/6 mouse zygotes via pronuclear microinjection. DNA from the 30 pups born was amplified using primers flanking *Mosmo* exon 1 and sequenced. One male founder that had a 386 bp deletion, including all of exon 1, was backcrossed with C57BL/6 females for two generations, then heterozygotes were intercrossed. *Mosmo* knockout mice were genotyped using two sets of primers to detect the wild-type or KO allele: Fwd (wild-type set 1), 5′-GATAAACTGACCATCATCTCAGGATG-3′; Rev (wild-type set 1), 5′-ACTTCAAAGGGGAAAGGGGGAG-3′; Fwd (wild-type set 2), 5′-GGGCGATGGATAAACTGACC-3′; Rev (wild-type set 2), 5′-CGCCTCTTTCTTGAGGACAC-3′; Fwd (mutant set 1), 5′-CCAGTTCCTTCCCATTGCATCT-3′; Rev (mutant set 1), 5′-GCAGTTCAAATACAAGACCGTTCC-3′; Fwd (mutant set 2), 5′-CCGAGAGCTGGGATTCGTAG-3′; and Rev (mutant set 2), 5′-CCACAGACACTTCAAAGGGGA-3′ (Fig. S2B).

### scRNA-seq analysis

We used single-cell transcriptome data from the *MouseGastrulationData* R package for mouse gastrulation at E7.5 and E8.5. The raw single cell RNA-seq data we analyzed can be found in ArrayExpress under accession number E-MTAB-6967. We used Seurat (version 3.2.3) to analyze the scRNA-seq data (Pijuan-Sala et al., 2019). Our workflow for processing the scRNA-seq data involves data pre-processing, centered log ratio transformation across features, scaling with a linear model, dimensionality reduction and visualization. The annotation of cell types was based on metadata labels included in the data. Cells were plotted based on their euclidean coordinates after a UMAP dimensionality reduction, and *Mosmo* expression is given in normalized read counts.

### Molecular modeling

The 184 amino acid M-Stem domain from human MEGF8 comprises amino acids 2463-2647, and is tightly sandwiched between an EGFL module and the hydrophobic transmembrane helix. The Hhpred program in the MPI bioinformatics toolkit (Gabler et al., 2020) was used to define the boundaries and β-sheet nature of this elusive domain by sequence and structural profile matching to MEGF8 orthologs, and Attractin (ATRN) and Attractin-like 1 (ATRNL1) paralogs. The structure of the isolated MEGF8 M-Stem domain (where a 32 residue, disordered Pro and Gly rich insert, amino acids 2530-2562, relative to a compact ATRN loop, was replaced by a Gly) was predicted and modeled by trRosetta based on its *de novo* folding algorithm in a template-free fashion, guided by deep learning-derived restraints of residue distances and orientations (Yang et al., 2020). The confidence of the predicted model is high with an estimated TM-score of 0.567. Similar folds present in other PDB structures were revealed by PDBeFOLD searches (Krissinel and Henrick, 2004) with the trRosetta-derived models of MEGF8 and related ATRN and ATRNL1 Stem domains. Residue conservation profiles were mapped to the Stem domain structure with the ConSurf program (Ashkenazy et al., 2016).

### Constructs

*MEGF8*-1D4, *MEGF8*ΔN-1D4, *MEGF8*ΔC-1D4, *Mgrn1*-3xFLAG, *Mgrn1^Mut1^*-3xFLAG, *Smo*-EGFP and *Mosmo*-1D4 have been previously described (Kong et al., 2020; Pusapati et al., 2018). *Mosmo*-3xHA was synthesized as a gBlock (Integrated DNA Technologies) and used as a template for the PCR amplification step. To generate MEGF8ΔN^Stem^, *MEGF8* (NM_001410.3) nucleotide sequence coding for amino acids 2313-2778 was PCR amplified using full-length *MEGF8* as a template. All constructs were cloned initially into the pENTR2B plasmid (Thermo Fisher Scientific, Invitrogen) and then transferred into pEF5/FRT/V5-DEST (Thermo Fisher Scientific, Invitrogen) or pLenti CMV PURO DEST (Campeau et al., 2009) using Gateway recombination methods (Thermo Fisher Scientific, Invitrogen).

### Reagents and antibodies

Recombinant SHH was expressed in bacteria and purified in the lab as previously described (Bishop et al., 2009). Briefly, His-tagged SHH-N (C24II followed by human SHH amino acids 25-193) was expressed in *Escherichia coli* [BL21 strain; Rosetta2 (DE3)pLysS]. Cells were lysed in 10 mM phosphate buffer (pH 7.5), 500 mM NaCl, 1 mM 2-mercaptoethanol, 1 mM PMSF and 1× protease inhibitor cocktail, followed by centrifugation at 20,000 ***g*** for 30 min at 4°C. Clarified samples were incubated with Ni-NTA resin (Qiagen) for 1 h at 4°C. The resin was washed with 20 column volumes of wash buffer A (lysis buffer without protease inhibitors), followed by wash buffer B (wash buffer A+10 mM Imidazole), and bound proteins were eluted with elution buffer (wash buffer A+250 mM Imidazole). Peak fractions were pooled, concentrated using a 5 kDa cut-off VIVASPIN 15R (Life Technologies), and loaded onto a Superdex 75 gel filtration column (Amersham Biosciences) equilibrated with column buffer [10 mM HEPES (pH 7.5), 150 mM NaCl and 1 mM DTT]. The recombinant protein was >98% pure, as assessed from Coomassie staining, and stored at −80°C. The selection antibiotic puromycin was purchased from MilliporeSigma. The transfection reagent XtremeGENE 9 was purchased from Roche Molecular Systems and polybrene from MilliporeSigma. Bafilomycin A1 was purchased from Cayman Chemical. Vismodegib and Bortezomib were purchased from LC labs. The following primary antibodies were purchased from the following vendors: mouse anti-1D4 (The University of British Columbia, 56504; 1:5000); rat anti-E-cadherin (clone ECCD-2, Thermo Fisher Scientific, 13-1900; 1:1000); mouse anti-FLAG (clone M2, MilliporeSigma, F1804; 1:2000); goat anti-GFP (Rockland Immunochemicals, 600-101-215; 1:1000); rabbit anti-GFP (Novus Biologicals, NB600-308; 1:5000); mouse anti-GLI1 (clone L42B10, Cell Signaling, 2643; 1:1000); mouse anti-HA (clone 2-2.2.14, Thermo Fisher Scientific, 26183; 1:2000); rabbit anti-p38 (Abcam, ab7952; 1:2000); and rabbit anti-RNF156 (anti-MGRN1, Proteintech, 11285-1-AP; 1:500); mouse anti-ɑ-Tubulin (Clone DM1A, MilliporeSigma, T6199; 1:10,000); mouse anti-acetylated-Tubulin (MilliporeSigma, T6793; 1:10,000). The following primary antibodies were generated in the lab or received as a gift: guinea pig anti-ARL13B (1:1000) (Dorn et al., 2012); rabbit anti-SMO (designed against an intracellular epitope, 1:2000) (Rohatgi et al., 2007); and rabbit anti-MEGF8 (1:2000) (Kong et al., 2020). Hoechst 33342 and secondary antibodies conjugated to horseradish peroxidase (HRP) or Alexa Fluor dyes were obtained from Jackson ImmunoResearch Laboratories and Thermo Fisher Scientific as follows: Peroxidase AffiniPure donkey anti-mouse IgG (Jackson ImmunoResearch Laboratories, 715-035-150, 1:10,000); Peroxidase AffiniPure donkey anti-rabbit IgG (Jackson ImmunoResearch Laboratories, 111-035-144, 1:10,000); Peroxidase AffiniPure donkey anti-goat IgG (Jackson ImmunoResearch Laboratories, 705-035-003, 1:10,000); donkey anti-rabbit IgG (H+L) Highly Cross-Adsorbed Secondary Antibody, Alexa Fluor 488 (Thermo Fisher Scientific, A-21206, 1:1000); donkey anti-rabbit IgG (H+L) Highly Cross-Adsorbed secondary antibody, Alexa Fluor 594 (Thermo Fisher Scientific, A-21207, 1:1000); donkey anti-mouse IgG (H+L) secondary antibody, Alexa Fluor 488 (Thermo Fisher Scientific, A-21202, 1:1000); donkey anti-mouse IgG (H+L) secondary antibody, Alexa Fluor 647 (Thermo Fisher Scientific, A-31571, 1:1000); Alexa Fluor 488 AffiniPure donkey anti-guinea Pig IgG (H+L) (Jackson ImmunoResearch Laboratories, 706-545-148, 1:1000); and Alexa Fluor 647 AffiniPure donkey anti-guinea pig IgG (H+L) (Jackson ImmunoResearch Laboratories, 706-605-148, 1:1000).

### Immunoprecipitation and western blotting

Whole-cell extracts from HEK293T and NIH/3T3 cells were prepared in immunoprecipitation (IP) lysis buffer: 50 mM Tris (pH 8.0), 150 mM NaCl, 1% NP-40, 0.25% sodium deoxycholate, 1 mM DTT and 1× SIGMAFAST protease inhibitor cocktail (MilliporeSigma). Cells were lysed for 1 h on a shaker at 4°C, supernatants were clarified by centrifugation (20,000 ***g*** for 30 min at 4°C), and 1D4 tagged MOSMO or MEGF8 was captured by a 1D4 antibody (The University of British Columbia) covalently conjugated to Protein A Dynabeads (Thermo Fisher Scientific, Invitrogen). Immunoprecipitates were washed once with IP Wash Buffer A [50 mM Tris (pH 8.0), 150 mM NaCl, 1% NP-40, 0.25% sodium deoxycholate and 1 mM DTT], once with IP Wash Buffer B [50 mM Tris (pH 8.0), 500 mM NaCl, 0.1% NP-40, 0.25% sodium deoxycholate and 1 mM DTT], and finally with IP Wash Buffer C [50 mM Tris (pH 8.0), 0.1% NP-40, 0.25% sodium deoxycholate and 1 mM DTT]. Proteins were eluted by resuspending samples in 2× NuPAGE LDS sample buffer (Thermo Fisher Scientific, Invitrogen) supplemented with 100 mM DTT, incubated at 37°C for 1 h and subjected to SDS-PAGE (Fig. 3D,F).

Whole-cell extracts were prepared in RIPA lysis buffer: 50 mM Tris (pH 8.0), 150 mM NaCl, 2% NP-40, 0.25% sodium deoxycholate, 0.1% SDS, 0.5 mM TCEP, 10% glycerol, 1× SIGMAFAST protease inhibitor cocktail (MilliporeSigma) and 1× PhosSTOP phosphatase inhibitor cocktail (Roche). The resolved proteins were transferred onto a nitrocellulose membrane (Bio-Rad Laboratories) using a wet electroblotting system (Bio-Rad Laboratories) followed by immunoblotting.

For the preparation of whole-embryo extracts, e12.5 embryos were collected and rinsed thoroughly in chilled PBS. Each embryo was then individually submerged in liquid nitrogen and pulverized using a mortar and pestle. The crushed tissue was then lysed in modified RIPA lysis buffer: 50 mM Tris (pH 8.0), 150 mM NaCl, 1% NP-40, 0.25% sodium deoxycholate, 0.1% SDS, 5 mM EDTA, 1 mM sodium fluoride, 1 mM sodium orthovanadate and 1× SIGMAFAST protease inhibitor cocktail (MilliporeSigma). The resolved proteins were then transferred onto a nitrocellulose membrane (Bio-Rad Laboratories) using a wet electroblotting system (Bio-Rad Laboratories) and immunoblotted.

### Cell surface biotinylation assay

Cell surface levels of MEGF8 (Fig. 3A) were determined by a biotinylation assay as described previously (Kong et al., 2020). Briefly, wild-type, *Mosmo^−/−^* and *Megf8^−/−^* NIH/3T3 cells were plated on 10 cm plates in Complete Medium. Once the cells were confluent, they were switched to Low Serum Medium for 24 h. On biotinylation day, the cells were removed from the 37°C incubator and placed on an ice-chilled metal rack in a 4°C cold room. The medium was removed and cells were quickly washed three times with ice-cold DPBS+ buffer (Dulbecco's PBS supplemented with 0.9 mM CaCl_2_, 0.49 mM MgCl_2_.6H_2_O, 5.6 mM dextrose and 0.3 mM sodium pyruvate). Biotinylation of cell surface proteins using a non-cell permeable and thiol-cleavable probe was initiated by incubating cells with 0.4 mM EZ-Link Sulfo-NHS-SS-Biotin (Thermo Fisher Scientific) in DPBS+ buffer for 30 min. Unreacted Sulfo-NHS-SS-Biotin was quenched with 50 mM Tris (pH 7.4) for 10 min. Cells were then washed three times with 1× Tris-buffered saline [25 mM Tris (pH 7.4), 137 mM NaCl and 2.7 mM KCl] and whole-cell extracts were prepared in Biotinylation Lysis Buffer A [50 mM Tris (pH 8.0), 150 mM NaCl, 2% NP-40, 0.25% sodium deoxycholate, 1x SIGMAFAST protease inhibitor cocktail (MilliporeSigma) and 1×PhosSTOP phosphatase inhibitor cocktail (Roche)]. Biotinylated proteins from clarified supernatants were captured on a streptavidin agarose resin (TriLink Biotechnologies), washed once with Biotinylation Lysis Buffer A, once with Biotinylation Wash Buffer A (Biotinylation Lysis Buffer A+0.5% SDS), once with Biotinylation Wash Buffer B (Biotinylation Wash Buffer A+150 mM NaCl) and finally once again with Biotinylation Wash Buffer A. Biotinylated proteins captured on streptavidin agarose resin were eluted in 1× NuPAGE-LDS sample buffer (Thermo Fisher Scientific, Invitrogen) containing 100 mM DTT at 37°C for 1 h and assayed by immunoblotting for MEGF8.

### Ubiquitylation assay

Ubiquitylation assays were performed as previously reported (Kong et al., 2020). Briefly, 8 million HEK293T cells were plated onto a 15 cm plate. 24 h after plating, the cells were transfected using PEI. 6 μg of each construct was transfected into the HEK293T cells at a DNA:PEI ratio of 1:3. An empty plasmid construct was used as filler DNA to ensure that each plate was transfected with the same amount of DNA. To enrich for ubiquitylated proteins, 36 h post-transfection, cells were pre-treated with 10 µM Bortezomib (a proteasome inhibitor) and 100 nM Bafilomycin A1 (a lysosome inhibitor) for 4 h. Cells were washed twice with chilled PBS and lysed in Ubiquitylation Lysis Buffer A comprising 50 mM Tris at pH 8.0, 150 mM NaCl, 2% NP-40, 0.25% sodium deoxycholate, 0.1% SDS, 6 M urea, 1 mM DTT, 10 µM Bortezomib, 100 nM Bafilomycin A1, 20 mM N-Ethylmaleimide (NEM, MilliporeSigma) and 1×SIGMAFAST protease inhibitor cocktail (MilliporeSigma). Clarified supernatants were diluted tenfold with Ubiquitylation Lysis Buffer B (Ubiquitylation Lysis Buffer A prepared without urea) to adjust the urea concentration to 600 mM. For these assays, we assessed ubiquitylation on GFP-tagged SMO. Ubiquitylated GFP tagged SMO (Fig. 3B,C) was captured using a GFP-binding protein (GBP) covalently conjugated to carboxylic acid decorated Dynabeads (Dynabeads M-270 carboxylic acid, Thermo Fisher Scientific). Immunoprecipitates were washed once with Ubiquitylation Wash Buffer A (Ubiquitylation Lysis Buffer B+0.5% SDS), once with Ubiquitylation Wash Buffer B (Ubiquitylation Wash Buffer A+1 M NaCl), and finally once again with Ubiquitylation Wash Buffer A. Proteins bound to dynabeads were eluted in 2× NuPAGE-LDS sample buffer (Thermo Fisher Scientific, Invitrogen) containing 30 mM DTT at 37°C for 1 h and assayed by immunoblotting.

### Immunofluorescence staining of cells, and tissue and image quantifications

Mouse tissue was prepared for immunofluorescence imaging as previously described (Kong et al., 2020). Briefly, mouse embryos of various ages were harvested and fixed in 4% (w/v) paraformaldehyde (PFA) in PBS at 4°C on a nutator. Fixation time varied depending on the age of the embryo (30 min for E8.5-E9.5, 1 h for E10.5-E11.5 and 2 h for E12.5). The embryos were then rinsed thoroughly in chilled PBS. To cryopreserve the tissue, the embryos were transferred to 30% sucrose in 0.1 M PB (pH 7.2) and allowed to equilibrate overnight. The embryos were then carefully dissected, then the desired tissues were mounted and frozen into Tissue-Plus OCT (optimal cutting temperature) compound (Thermo Fisher Scientific) and 12-14 µm sections were collected on a Leica CM1800 cryostat. Prior to staining, the tissues were blocked for 1 h at room temperature in immunofluorescence (IF) Blocking Buffer: 1% normal donkey serum (NDS) and 0.1% Triton-X diluted in PBS. In a humidified chamber, the sections were then incubated with primary antibodies overnight prepared in IF Blocking Buffer at 4°C, rinsed three times in PBST (PBS+0.1% Triton-X), incubated with secondary antibodies and Hoechst prepared in IF Blocking Buffer for 1 h at room temperature, rinsed three times in PBST, and then mounted in ProLong Gold antifade mountant (Thermo Fisher Scientific, Invitrogen).

NIH/3T3 cells and pMEFs were fixed in chilled 4% (w/v) PFA in PBS for 10 min and then rinsed thoroughly with chilled PBS. Cells were incubated in IF Blocking Buffer for 30 min, primary antibodies for 1 h and secondary antibodies for 30 min. Coverslips were mounted in ProLong Gold antifade mountant (Thermo Fisher Scientific, Invitrogen).

Fluorescent images were acquired on an inverted Leica SP8 confocal microscope equipped with a 63× oil immersion objective (NA 1.4). *Z*-stacks (∼4 µm sections) were acquired with identical acquisition settings (laser power, gain, offset, frame and image format) within a given experiment. An 4-8× optical zoom was used for imaging cilia to depict representative images. For the quantification of SMO at cilia, images were opened in Fiji (Schindelin et al., 2012) with projections of the maximum fluorescent intensities of *z*-stacks. Ciliary masks were constructed based on ARL13B images and then applied to corresponding SMO images to measure the fluorescence intensity of SMO at cilia.

### Vismodegib dosing via oral gavage

Vismodegib treatment was performed as described previously (Heyne et al., 2015). Briefly, *Mosmo^+/−^*×*Mosmo^+/−^* and wild-type×*Gli1^lacz/+^* mouse crosses were set up and monitored daily. Time E0 was defined as midnight prior to the visualization of the copulation plug. Female mice were weighed at ∼E0.25 (the morning the plug was visualized) and ∼E7.25. Only mice that gained 1.75 g over 7 days were deemed ‘likely pregnant’ and treated with either vehicle or vismodegib. For vismodegib treatment, a 3 mg/ml vismodegib solution was prepared in 0.5% methyl cellulose (MilliporeSigma) with 0.2% Tween. Vismodegib (40 mg/kg) was administered via oral gavage every 12 h (∼7am and 7pm) for a total of 3 days (E8.25-E11.25) or 4 days (E7.25-E11.25) (Figs 4C, 5A-D and Table S4). Embryos were harvested at E14.5, fixed in 4% (w/v) PFA in PBS for at least 24 h and then analyzed.

### Mouse embryo phenotyping analysis

Mouse embryo phenotyping was performed as described previously (Kong et al., 2020). Briefly, mouse embryos (E14.5) were fixed in 4% (w/v) PFA in PBS for at least 24 h. Necropsy was performed to determine visceral organ situs (i.e. lung and liver lobation, heart directionality, and positioning of the stomach, spleen and pancreas) (Table 1, Tables S2 and S4). For analysis by episcopic confocal microscopy (ECM), the samples were embedded in paraffin and processed as previously described (Liu et al., 2013). Briefly, the tissue block was sectioned using a Leica sledge microtome and serial images of the block face were captured with a Leica confocal microscope. The serial two-dimensional (2D) image stacks generated were then three-dimensionally (3D) reconstructed using Osirix software (Rosset et al., 2004) and digitally resliced in different orientations to aid in the analysis of intracardiac anatomy and the diagnosis of congenital heart defects (Liu et al., 2013) (Figs 1F and 5A).

### Whole-mount skeletal staining

Whole-mount skeletal stains were prepared using a modified published protocol (McLeod, 1980; Rigueur and Lyons, 2014). Briefly, E15.5-E16.5 mouse embryos were harvested and the skin and internal organs were removed to facilitate tissue permeabilization. The embryos were then fixed first in 95% ethanol overnight at room temperature followed by 100% acetone overnight at room temperature. To stain the cartilage, the embryos were incubated overnight at room temperature in 0.03% (w/v) Alcian Blue 8GX (Millipore Sigma) prepared in a solution of 80% ethanol and 20% glacial acetic acid. After visually confirming that the embryos were completely blue, the embryos were destained in a series of ethanol washes (2-3 h in 100% ethanol, 75% ethanol, 50% ethanol and 25% ethanol). To stain the bone, the embryos were then incubated overnight at 4°C in 0.005% (w/v) Alizarin Red S (Millipore Sigma) prepared in 1% potassium hydroxide (KOH). The tissue was then cleared in 0.3% KOH for 1 to 3 days (changing the solution every day). Once the embryos cleared to the desired amount, the 0.3% KOH was replaced with glycerol. The embryos were transitioned through a series of glycerol solutions (20% for 1 day, 50% for 1 day and then 80% for 1 day). The skeletons were then kept in 80% glycerol for prolonged storage.

### Whole-mount lung staining and branching analysis

Whole-mount lungs were prepared as previously described (Metzger et al., 2008). Briefly, E12.5 mouse embryos were harvested and the lungs were carefully excised. The lungs were fixed in 4% (w/v) PFA in PBS for 1 h and then rinsed thoroughly in PBS at room temperature. The lungs were dehydrated in a series of methanol washes: once in 25% methanol/PBS (v/v), once in 50% methanol/PBS, once in 75% methanol/PBS and twice in 100% methanol. The dehydrated lungs were then bleached for 15 min in 5% H_2_O_2_/methanol at room temperature and then rehydrated in a series of PBT washes (PBS with 0.1% Tween-20): once in 75% methanol/PBS, once in 50% methanol/PBT, once in 25% methanol/PBT and thrice in 100% PBT. The lungs were blocked for 1 h at room temperature in Whole-mount (WM) Blocking Buffer: 5% donkey serum and 0.5% Triton X-100 diluted in PBS. For primary antibody labeling, the lungs were incubated overnight at 4°C in rat anti-E-cadherin antibody (clone ECCD-2, Thermo Fisher Scientific, 13-1900) diluted 1:1000 in WM Blocking Buffer. The following day, the lungs were thoroughly rinsed in PBT (8×30 min). For secondary antibody labeling, the lungs were incubated overnight at 4°C in biotin-conjugated donkey anti-rat IgG (Jackson ImmunoResearch Laboratories) diluted 1:250 in WM Blocking Buffer. The lungs were then thoroughly rinsed in PBT (8×30 min), the biotin was visualized using the VECTASTAIN Elite ABC Kit (Vector, PK-16100) and the signal was amplified using a Tyramide Signal Amplification System (Cy3, Perkin Elmer). Stained lungs were mounted in Vectashield with DAPI (Vector) and imaged on a Thunder Imager Model Organism (Leica) (Fig. 1E and Fig. S3A).

### Whole-mount β-galactosidase staining of mouse embryos

Mouse embryos were processed for β-galactosidase staining using a modified published protocol (Nagy et al., 2007). Briefly, embryos were harvested from *Mosmo^+/−^*×*Mosmo^+/−^*;*Gli1^lacZ/+^* mouse crosses and fixed at 4°C in 4% (w/v) PFA in PBS for varying durations of time depending on their age (E8.5 and E9.5 for 10 min, E10.5 for 12 min, E11.5 for 15 min and E12.5 for 20 min). The embryos were then rinsed thoroughly in PBS and permeabilized for either 2 h (≤E9.5) or overnight (≥E10.5) at 4°C in permeabilization solution: 0.02% sodium deoxycholate and 0.01% NP-40 diluted in PBS. Following permeabilization, the embryos were placed in staining solution: 1 mg/ml X-gal (Thermo Fisher Scientific), 2 mM MgCl_2_, 0.02% NP-40, 5 mM potassium ferricyanide, 5 mM potassium ferrocyanide, 0.01% sodium deoxycholate diluted in 0.1 M phosphate buffer (pH 7.2). The embryos were stained for 2 h at 37°C. To remove residual yellow color from the staining solution, the embryos were rinsed in permeabilization solution (2×15 min). The embryos were fixed overnight in 4% (w/v) PFA in PBS at 4°C, rinsed in PBS, and then imaged.

### *In situ* hybridization of whole-mount and sectioned tissue

As previously described (Pusapati et al., 2018), to generate a *Mosmo in situ* probe, *Mosmo* specific primers were designed using the program Primer3: forward 5′-acacgtgtgtgctgaaaagc-3′ and reverse 5′-gagattaaccctcactaaagggatgagcaggtaacccatctcc-3′. The underlined sequence marks the T3 polymerase binding site that was incorporated into the reverse primer. The *Mosmo* probe was generated using a digoxigenin (DIG) RNA Labeling Kit (Roche). Briefly, the probe was generated from the *in vitro* transcription of PCR products amplified from mouse neural progenitor cell cDNA. After overnight hybridization at 65°C, the signal was visualized using Anti-DIG-alkaline phosphatase (AP) Fab fragments (Roche) and NBT/BCIP (Roche).

### Quantification and statistical analysis

Most data were analyzed using GraphPad Prism 9. Violin plots (Fig. 2D and Fig. S4A) were created using the ‘Violin plot (truncated)’ appearance function. In Prism 9, the frequency distribution curves of the violin plots are calculated using kernal density estimation. By using the ‘truncated’ violin plot function, the frequency distributions shown are confined within the minimum to maximum values of the data set. On each violin plot, the median (central bold line) and quartiles (adjacent thin lines, representing the first and third quartiles) are labeled. In Prism 9, the statistical significance between two groups was determined using either Mann–Whitney (Fig. 2D) or an unpaired *t*-test (Fig. 6C,D) and the significance between three or more groups was determined using the Kruskal–Wallis test (Fig. 4C, Fig. S4A). For each of these figures, *P*-values were calculated using Prism 9 and reported in the figure legend using the following key: not-significant (ns)>0.05, *P<0.05, **P<0.01, ***P<0.001 and ****P<0.0001 Additional figure details regarding the *n* value and statistical test applied are reported in the individual figure legends.

Disruptions in offspring viability due to genotype (Fig. 1A and Table S1) or treatment (Fig. 5D and Table S5) were determined using the chi-squared test. Briefly, *Mosmo^+/−^*×*Mosmo^+/−^* crosses were set up, live embryos were collected and deviation from the expected Mendelian ratio of 1:2:1 was calculated [not-significant (ns)>0.05, **P<0.01 and ***P<0.001].

All cell biological and biochemical experiments were performed two to three independent times, with similar results. To validate *Mosmo^−/−^* primary mouse embryonic fibroblasts (pMEFs), three independent cell lines were generated (each from a single embryo) and compared against control (*Mosmo^+/+^* and *Mosmo^+/−^*) pMEFs generated from embryos within the same litter (Fig. 2A). Similarly, two whole embryo lysate samples were prepared (each from a single *Mosmo^−/−^* embryo) and compared against control (*Mosmo^+/+^* and *Mosmo^+/−^*) lysates prepared from embryos within the same litter (Fig. 2C).

## Supplementary Material

Supplementary information
