# Supplementary material for: Gene-teratogen interactions influence the penetrance of birth defects by altering Hedgehog signaling strength
Source: Development. 2021 Oct 4;148(19):dev199867. doi: 10.1242/dev.199867 (PMC8513608; doi:10.1242/dev.199867)
Supplement: Supplementary information [file develop-148-199867-s1.pdf]

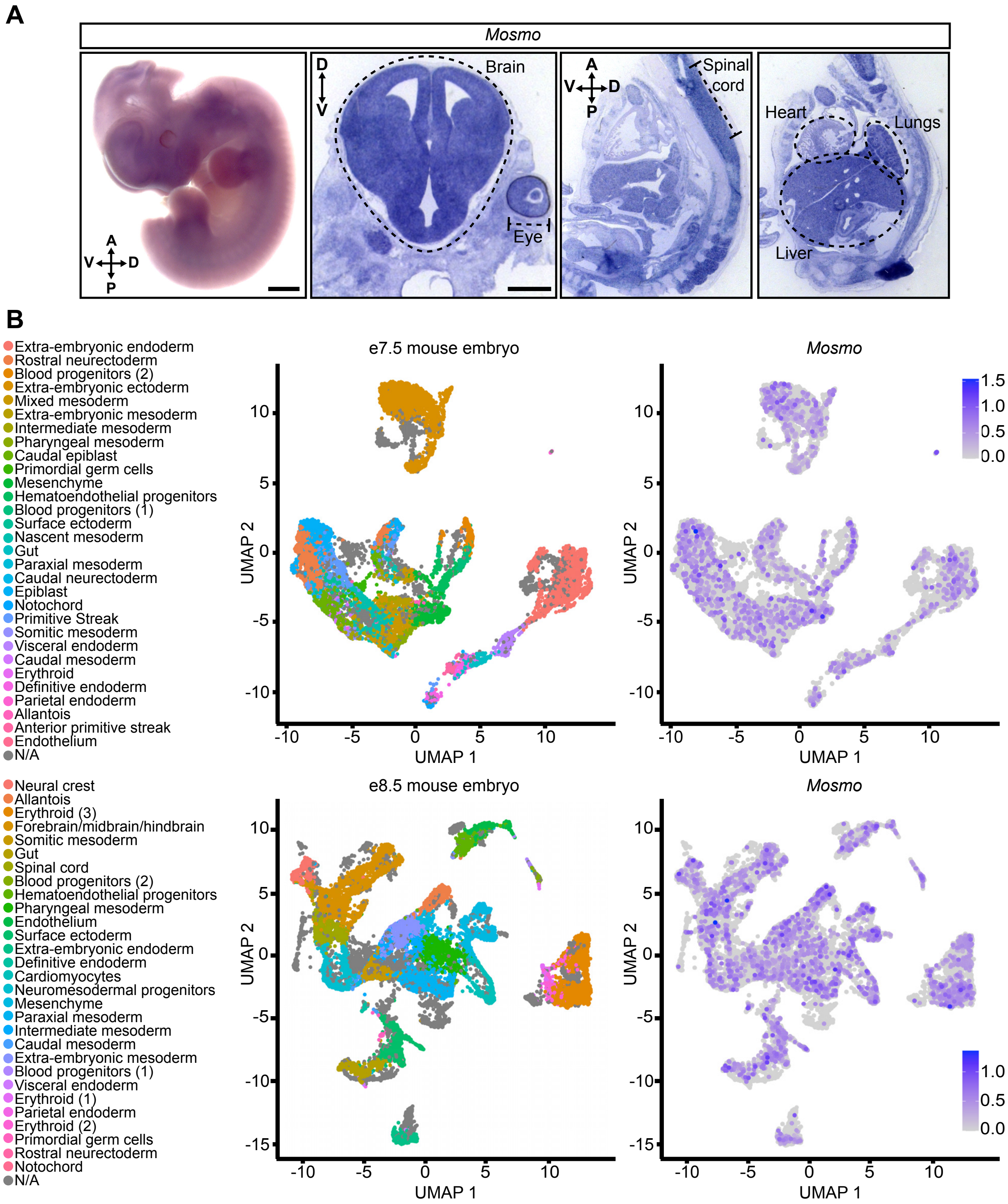

Fig. S1. *Mosmo* is widely expressed in the developing embryo

(A) Whole-mount *in situ* hybridization was used to assess *Mosmo* expression in whole-mount mouse embryo preparations (e11.5, leftmost panel) or in coronal brain and sagittal whole body sections (e13.5, three right panels). Scale bars are 1 mm. **(B)** UMAP analysis of previously published single-cell RNAseq data (Pijuan-Sala et al., 2019) showing *Mosmo* expression in an abundance of single cells from e7.5 (top) and e8.5 (bottom) embryos.

Development • Supplementary information

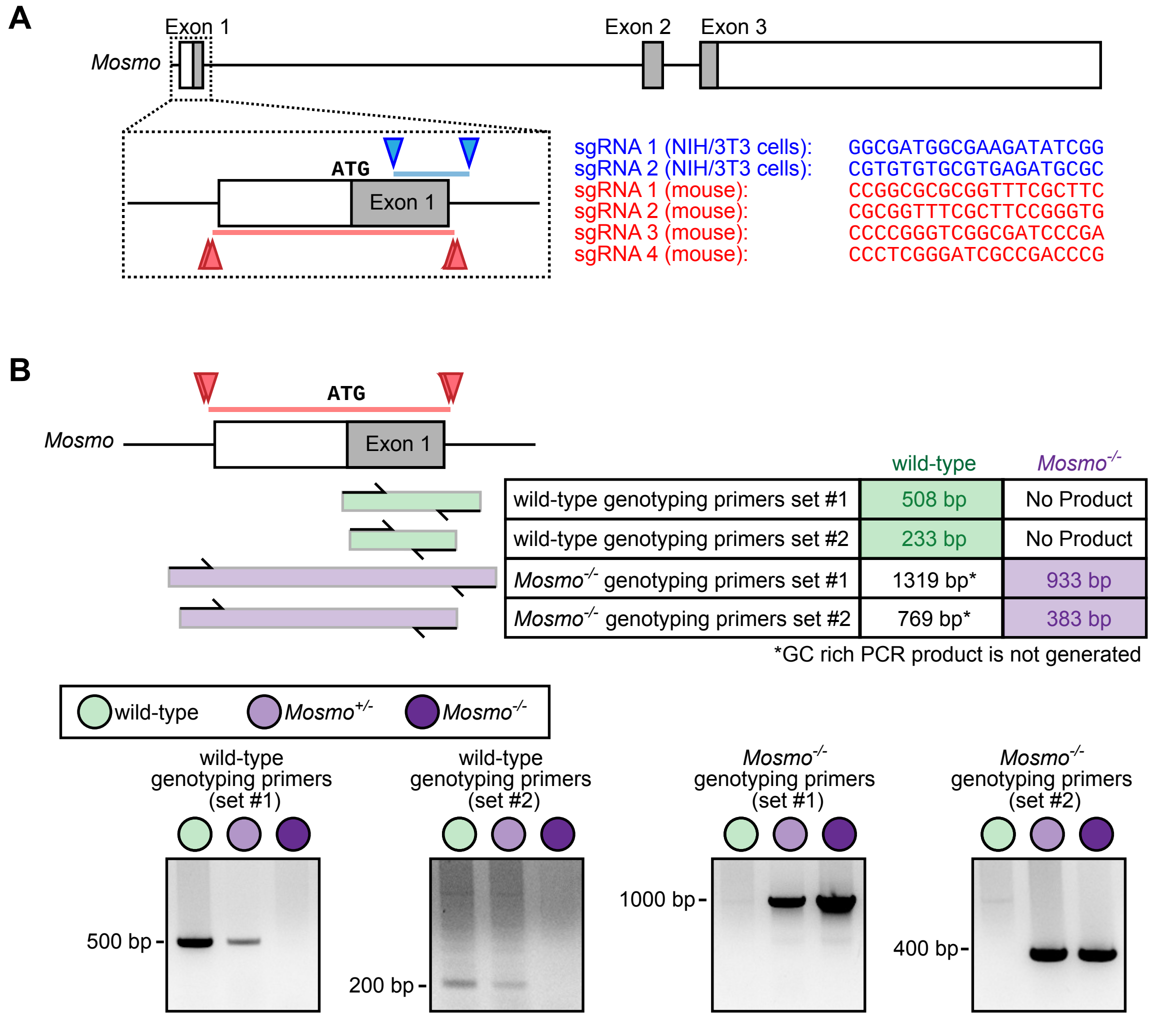

Fig. S2. Construction of *Mosmo*$^{-/-}$ mice using CRISPR/Cas9 mediated genome editing (A) *Mosmo* knockout (KO) strategy in NIH/3T3 cells and mice. Schematic of the *Mosmo* gene with exons represented as boxes, introns represented as a line, and the coding regions shaded in gray (top). Exon 1 is enlarged (below) with arrows marking the sgRNA guide targets. Guide sequences, targets, and deleted regions in NIH/3T3 cells and mouse embryos are shown in blue and red respectively. **(B)** PCR genotyping strategy to distinguish between wild-type and KO alleles. The *Mosmo*$^{-/-}$ mouse has a 386 bp deletion (red line) that includes a removal of the entire first exon (white and gray box). Exon 1 is GC rich and thus a combination of four genotyping primers (located within and outside of the deleted region) were used to determine if the allele has the 386 bp deletion. Representative images of the genotyping PCR are shown below.

Development • Supplementary information

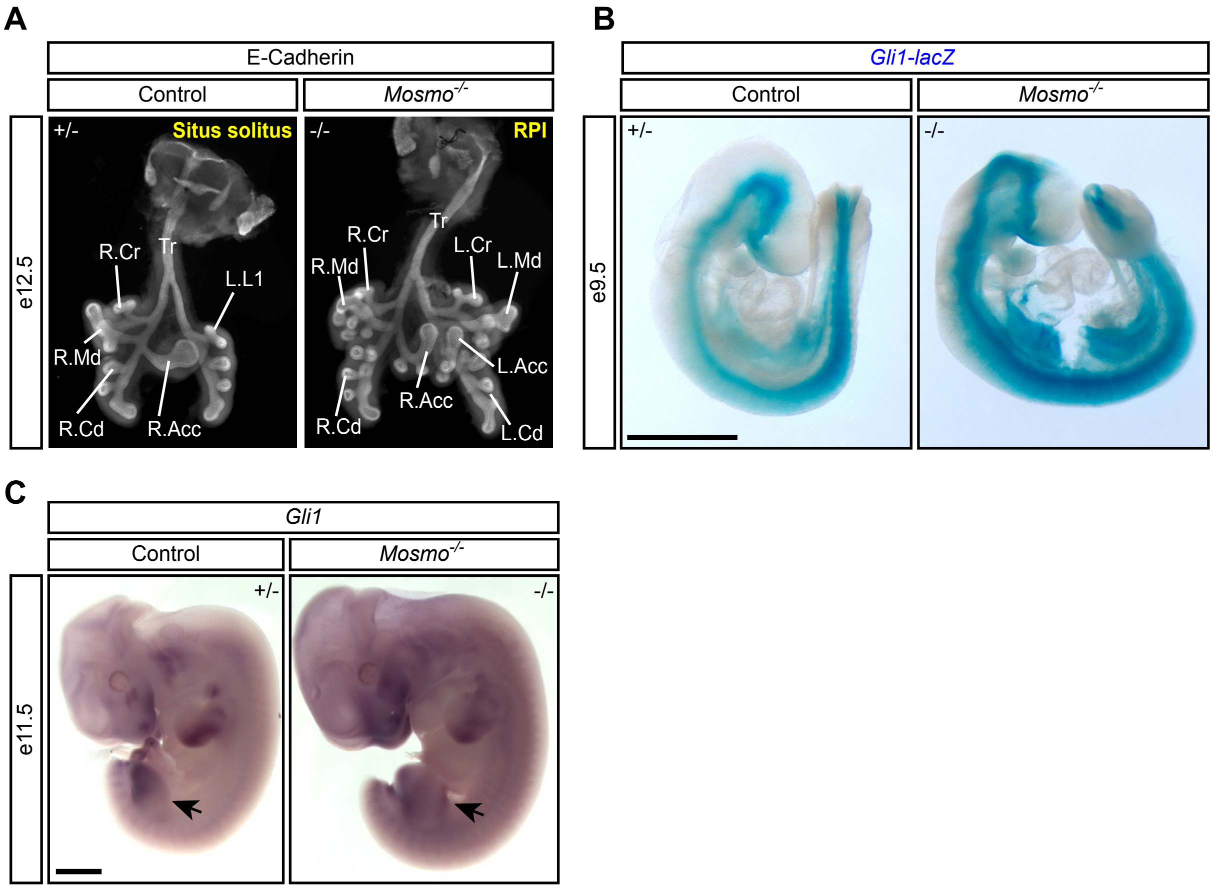

Fig. S3. Developmental phenotypes in *Mosmo*$^{-/-}$ embryos

(A) Whole-mount lungs (ventral view) of e12.5 control (*Mosmo*$^{+/-}$) and *Mosmo*$^{-/-}$ embryos immunostained for E-cadherin to show the airway epithelium. Normal mouse lungs have one lobe on the left (L.L1) and four lobes on the right (R.Acc, right accessory; R.Cr, right cranial; R.Cd, right caudal; R.Md, right middle). *Mosmo*$^{-/-}$ lungs exhibit right pulmonary isomerism (RPI), a duplication of the right lung morphology on the left side (L.Acc, left accessory; L.Cd, left caudal; L.Cr, left cranial; L.Md, left middle). **(B)** Whole-mount e9.5 control (*Mosmo*$^{+/-}$; *Gli1*$^{lacZ/+}$) and knockout (*Mosmo*$^{-/-}$; *Gli1*$^{lacZ/+}$) littermates stained with X-gal to visualize *Gli1-lacZ* expression. Scale bar is 1 mm. **(C)** Whole-mount in situ hybridization was used to assess *Gli1* expression in e11.5 control (*Mosmo*$^{+/-}$) and *Mosmo*$^{-/-}$ littermates. Arrows denote areas of elevated Hh signaling activity in the anterior hindlimb. Scale bar is 1 mm.

Development • Supplementary information

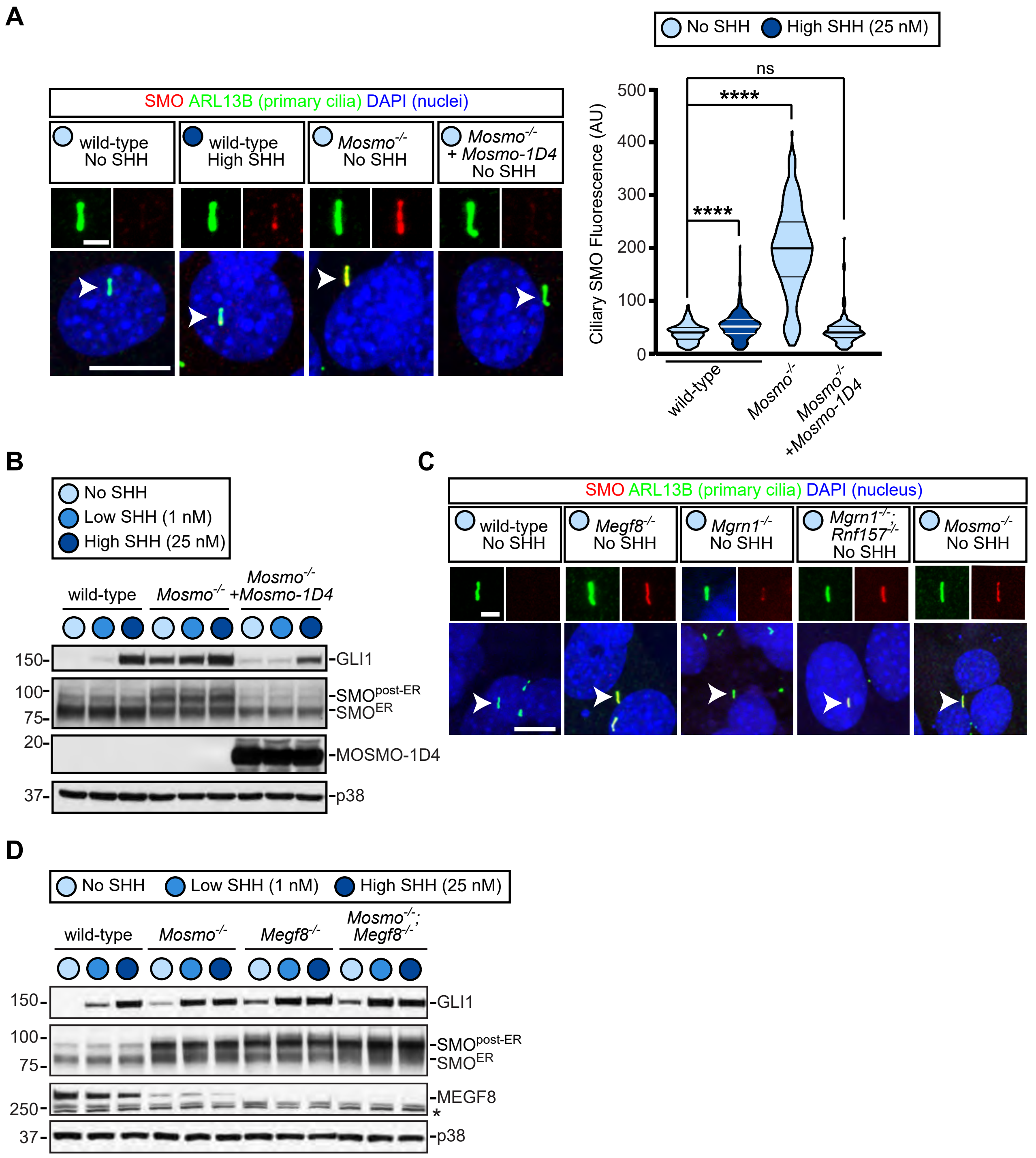

Development • Supplementary information

Fig. S4. Signaling defects in *Mosmo*$^{-/-}$ NIH/3T3 can be corrected by stable re-expression of Mosmo-1D4

(A-B) Ciliary SMO abundance in the indicated NIH/3T3 cell lines: wild-type (no SHH n=213, high SHH n=299), *Mosmo*$^{-/-}$ (n=239), or *Mosmo*$^{-/-}$ with stable re-expression of Mosmo-1D4 (n=156). Confocal fluorescence microscope images (left) of SMO (red) accumulation at primary cilia (ARL13B, green) in various NIH/3T3 cell lines. Arrowheads identify individual cilia captured in zoomed images above each panel. Nuclei were labeled with DAPI (blue). Ciliary SMO abundance was measured and violin plots generated (right), with horizontal lines denoting the median and interquartile ranges. Statistical significance was determined using the Kruskal-Wallis test (ns>0.05, ****P<0.0001). Scale bars, 10 μm in merged panels and 2 μm in zoomed displays. **(B)** Immunoblots showing GLI1 (as a measure of Hh signaling strength) and SMO abundance in NIH/3T3 cell lines treated with either no, low (1 nM), or high (25 nM) concentrations of SHH. Two populations of SMO are labeled, one localized in the endoplasmic reticulum (ER) and the other localized in post-ER compartments. p38 was used as a loading control. **(C)** Confocal fluorescence microscopy images of SMO (red) localized to the primary cilium (ARL13B, green) in the indicated NIH/3T3 cell lines. Nuclei were labeled with DAPI (blue). Scale bars, 10 μm in merged panels and 2 μm in zoomed displays. **(D)** Immunoblots of GLI1, SMO, and MEGF8 in the indicated NIH/3T3 cell lines treated with varying concentrations of SHH. p38 was used as a loading control. *indicates non-specific bands.

Development • Supplementary information

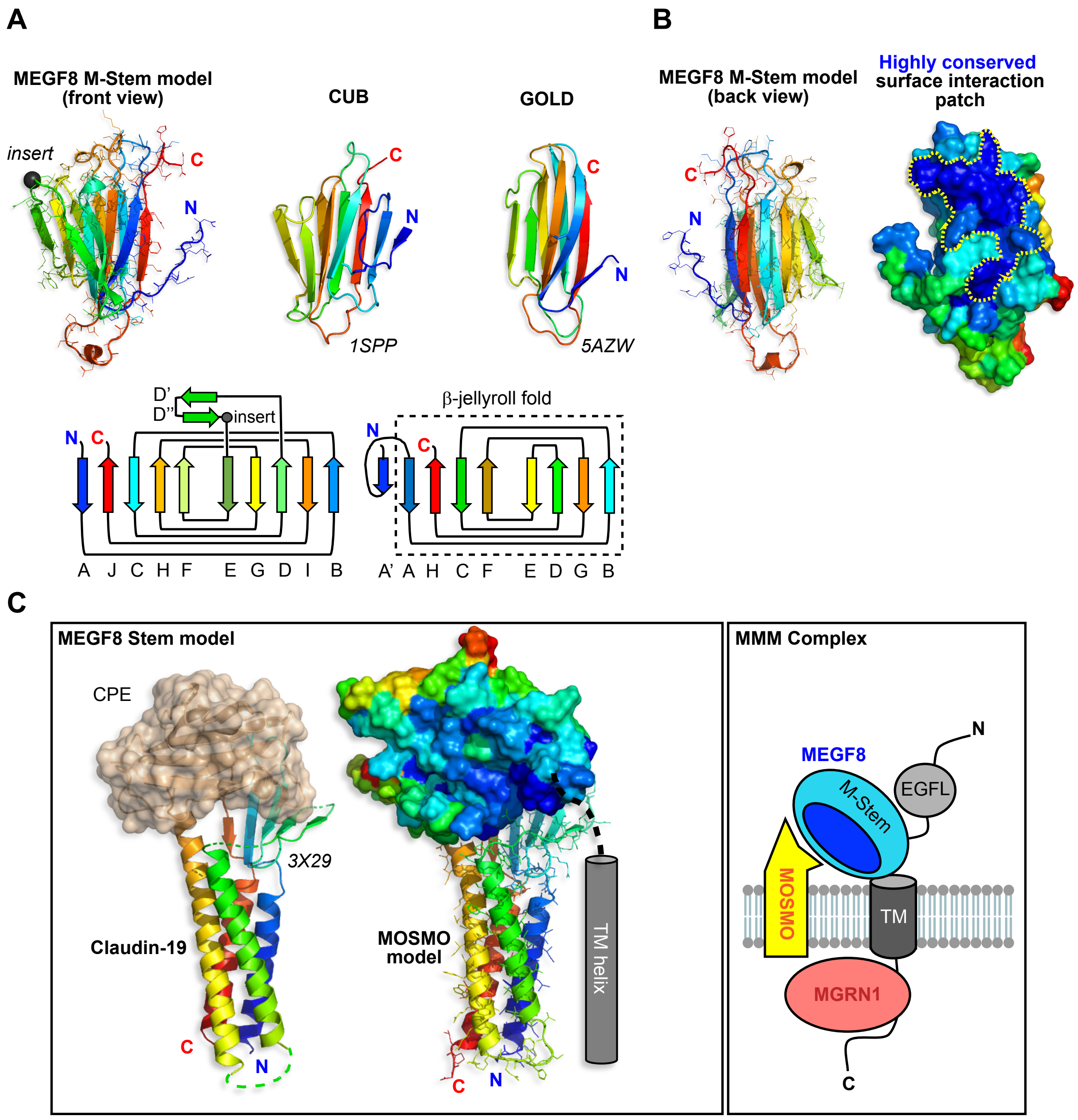

Development • Supplementary information

Fig. S5. A model of the interaction between the M-Stem domain of MEGF8 and MOSMO. **(A)** Molecular model of the MEGF8 M-Stem domain (see **Figure 3F**) predicted by trRosetta is shown in cartoon form with side chains, the chain color ramped from blue (N-terminus) to red (C-terminus). An unstructured 32 amino acid disordered segment (labeled “insert”) was replaced with a glycine residue (black C sphere) before modelling. Shown to its right are PDBeFOLD superposed CUB and GOLD domains (respectively from PDB files 1SPP and 5AZW) and below are ‘open book’ topology depictions of MEGF8 M-Stem (left) and the CUB domain (right, with the dotted line showing the β-jellyroll fold overlap with the GOLD domain) with β-strands labeled below. In the M-Stem domain, we highlight the D’-D” β-hairpin that sprouts between β-strands D and E, and the position of the 32 amino acid unstructured insert (black ball) is noted in the D”-E loop. **(B)** If we rotate the MEGF8 M-Stem domain model by ~180°, revealing the ‘back’ β-sheet face (that is free of the D’-D” β-hairpin overhang) and show the surface conservation profile calculated by ConSurf, a highly conserved interaction epitope in dark blue is visible. **(C)** A molecular model of the MOSMO fold was predicted by trRosetta using Claudin structure templates drawn from the PDB and integrating this information with the deep-learning-derived distance and orientation restraints to calculate the final model. To the left of the Claudin-based model for MOSMO is the structure of Claudin-19 docked to the Clostridium perfringens enterotoxin C-terminal domain (CPE, with a β-jellyroll fold related to CUB) from PDB file 3X29. This CPE-Claudin-19 complex offers a template for the interaction of MEGF8’s M-Stem domain with MOSMO that utilizes the conserved surface patch (colored dark blue in B) in the β-sheet formed by the J, C, H and F strands (see A) of the M-Stem CUB-like fold. **(D)** A schematic of the MMM complex. The MEGF8 M-Stem-MOSMO and MEGF8-MGRN1 interactions have been experimentally validated (**Figures 3E-3G** and (Kong et al., 2020)).

Development • Supplementary information

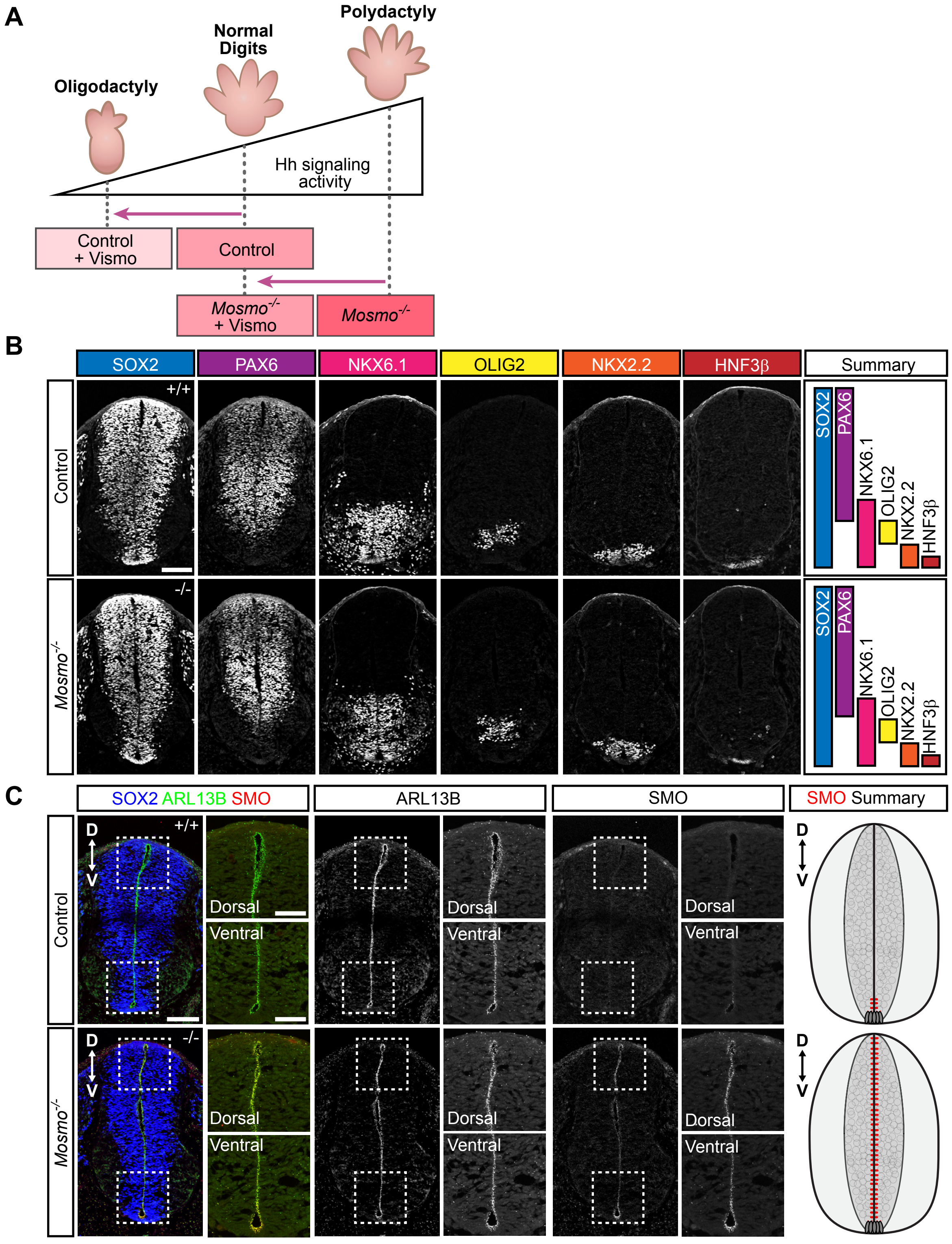

Fig. S6. Analysis of spinal cord patterning in control and Mosmo$^{-/-}$ embryos

(A) A proposed model for how a combination of genotype and SMO inhibition by vismodegib influences Hh signaling strength and consequently digit number in developing embryos. **(B-C)** Representative images of transverse sections of e10.5 control (*Mosmo*$^{+/+}$ and *Mosmo*$^{+/-}$) and *Mosmo*$^{-/-}$ spinal cords. **(B)** Distribution of transcription factors within the developing spinal cord. Scale bar is 100 µm. **(C)** Distribution of SMO (red) within the primary cilia (ARL13B, green) of spinal neural progenitors (SOX2, blue). Scale bars, 100 µm in merged panels and 50 µm in zoomed displays. Quantification available in **Figure 6C**.

Development • Supplementary information

Table S1. Related to Figure 1: Genotypes of live offspring derived from *Mosmo*$^{+/-}$ intercrosses.

Age	*Mosmo*$^{+/+}$	*Mosmo*$^{+/-}$	*Mosmo*$^{-/-}$	Total	Chi square p-value	Average Litter Size
Expected	*25%*	*50%*	*25%*			
e9.5-e13.5	17 (19.8%)	43 (50%)	26 (30.2%)	n = 86 (9 litters)	p = 0.390	9.6
e14.5-e18.5	24 (32.4%)	46 (62.2%)	4 (5.4%)	n = 74 (13 litters)	p = 0.000503	5.7
P0-P18	12 (28.6%)	30 (71.4%)	0 (0%)	n = 42 (8 litters)	p = 0.000685	5.3

Significant deviation from the expected Mendelian ratio of 1:2:1 was calculated using the chi-square test (ns>0.05 and ***P<0.001).

Table S2. Related to Figure 1 and 4: Heart, visceral organ situs, and digit number in wild-type (*Mosmo*$^{+/+}$), *Mosmo*$^{-/-}$, and *Mosmo*$^{-/-}$;*Megf8*$^{m/m}$ mouse embryos.

Mosmo$^{+/+}$ embryos

Embryo ID	Body situs	Heart	VA Alignment	Septal Defects	Aortic Arch	Lung Situs	Abdomen Situs	Digitation
1	SS	Lev	CC	None	LAA	4R:1L	L(St/Sp/P)	Normal
2	SS	Lev	CC	None	LAA	4R:1L	L(St/Sp/P)	Normal
3	SS	Lev	CC	None	LAA	4R:1L	L(St/Sp/P)	Normal

***Mosmo*$^{-/-}$ embryos**

Embryo ID	Body situs	Heart	VA Alignment	Septal Defects	Aortic Arch	Lung Situs	Abdomen Situs	Digitation
1	HTX	Mes	TGA	AVSD	LAA	3R:3L:1M	L(St/Sp/P)	PDD
2	HTX	Lev	PTA	AVSD	LAA	3R:3L:1M	L(St/Sp/P)	PDD
3	HTX	Dex	TGA	AVSD	RAA	3R:3L:1M	L(St/Sp/P)	PDD
4	HTX	Mes	TGA	AVSD	RAA	3R:3L:1M	L(St/Sp/P)	PDD
5	HTX	Dex	TGA	AVSD	RAA	1R:4L	L(St/Sp/P)	PDD
6	SIT	Dex	TGA	AVSD	RAA	1R:4L	R(St/Sp/P)	PDD
7	HTX	Dex	DORV	AVSD	RAA	3R:3L:1M	R(St/Sp/P)	PDD
8	HTX	Lev	TGA	AVSD	LAA	3R:3L:1M	L(St/Sp/P)	PDD
9	HTX	Dex	TGA	AVSD	LAA	3R:3L:1M	L(St/Sp/P)	PDD
10	SIT	Dex	TGA	AVSD	RAA	1R:4L	R(St/Sp/P)	PDD
11	HTX	Lev	PTA	AVSD	LAA	3R:3L:1M	L(St/Sp/P)	PDD

***Mosmo*$^{-/-}$;*Megf8*$^{m/m}$ double mutant embryos**

Embryo ID	Body situs	Heart	VA Alignment	Septal Defects	Aortic Arch	Lung Situs	Abdomen Situs	Digitation
1	HTX	Dex	PTA	-	RAA	3R:3L:1M	R(St/Sp/P)	PDD
2	HTX	Dex	TGA	AVSD	RAA	3R:3L:1M	R(St/Sp/P)	PDD
3	HTX	Dex	TGA	AVSD	RAA	3R:3L:1M	R(St/Sp/P)	PDD
4	HTX	Dex	DORV	VSD	LAA	3R:3L:1M	L(St/Sp/P)	PDD
5	HTX	Dex	TGA	AVSD	RAA	3R:3L:1M	R(St/Sp/P)	PDD

AVSD, atrioventricular septal defect; CC, concordant ventriculoarterial alignment; Dex, dextrocardia; DORV, double outlet right ventricle; HAA, hypoplastic arch; HTA, hypoplastic transverse heart; HTX, heterotaxy; IAA, interrupted aortic arch; LAA, left aortic arch; Lev, levocardia; L(St/Sp/P), Left sided stomach, spleen, pancreas; Mes, mesocardia; N/A: no digit data collected; NML, normal digit pattern; PDD, preaxial digit duplication; PTA, Persistent truncus arteriosus; RAA, right aortic arch; RPI, right pulmonary isomerism; R(St/Sp/P), Right sided stomach, spleen, pancreas; SIT, situs inversus; SS, situs solitus; SymLiv, symmetric liver; TGA, transposition of the great arteries; VA, ventriculoarterial; VSD, ventricular septal defect; VSD(Ao), ventricular septal defect located below the aorta; 1L, one lung lobe on left side; 4R, 4 lung lobes on right side.

Table S3. Related to Figure 1: Lung branching analysis in e12.5 mouse embryos

Genotype	Total embryos analyzed	Situs solitus	Right pulmonary isomerism (RPI)		
			Complete RPI	Partial RPI	
				Extra “right” branches on the left	Situs inversus
Mosmo$^{+/+}$	n = 2	n = 2/2 (100%)	n = 0/2 (0%)	n = 0/2 (0%)	n = 0/2 (0%)
Mosmo$^{+/-}$	n = 14	n = 14/14 (100%)	n = 0/14 (0%)	n = 0/14 (0%)	n = 0/14 (0%)
Mosmo$^{-/-}$	n = 5	n = 0/5 (0%)	n = 3/5 (60%)	n = 1/5 (20%)	n = 1/5 (20%)

Table S4. Related to Figure 5: Heart, visceral organ situs, and digit number in control (*Mosmo*$^{+/+}$ and *Mosmo*$^{+/-}$) and *Mosmo*$^{-/-}$ embryos treated with either vehicle or vismodegib.

Mosmo$^{+/+}$ embryos with vehicle treatment

Embryo ID	Body situs	Heart	VA Alignment	Septal Defects	Aortic Arch	Lung Situs	Abdomen Situs	Digitation
1	SS	Lev	CC	None	LAA	4R:1L	L(St/Sp/P)	Normal
2	SS	Lev	CC	None	LAA	4R:1L	L(St/Sp/P)	Normal
3	SS	Lev	CC	None	LAA	4R:1L	L(St/Sp/P)	Normal

***Mosmo*$^{-/-}$ embryos with vehicle treatment**

Embryo ID	Body situs	Heart	VA Alignment	Septal Defects	Aortic Arch	Lung Situs	Abdomen Situs	Digitation
1	HTX	Dex	TGA	AVSD	RAA	3R:3L:1M	R(St/Sp/P)	PDD
2	HTX	Lev	TGA	AVSD	LAA	3R:3L:1M	L(St/Sp/P)	PDD
3	HTX	Lev	TGA	AVSD	LAA	3R:3L:1M	L(St/Sp/P)	PDD
4	HTX	Dex	TGA	AVSD	RAA	3R:3L:1M	R(St/Sp/P)	N/A
5	HTX	Dex	TGA	AVSD	RAA	3R:3L:1M	R(St/Sp/P)	N/A
6	HTX	Lev	PTA	AVSD	LAA	3R:3L:1M	L(St/Sp/P)	N/A

Mosmo$^{+/+}$ and Mosmo$^{+/-}$ embryos with 3 Day and 4 Day vismodegib treatment

Embryo ID	Body situs	Heart	VA Alignment	Septal Defects	Aortic Arch	Lung Situs	Abdomen Situs	Digitation
1	SS	Lev	PTA	AVSD	LAA	N/A	L(St/Sp/P)	Oligo
2	SS	Lev	PTA	AVSD	RAA	N/A	L(St/Sp/P)	Oligo
3	SS	Lev	PTA	AVSD	LAA	N/A	L(St/Sp/P)	Oligo
4	SS	Lev	PTA	AVSD	LAA	N/A	L(St/Sp/P)	Oligo
5	SS	Lev	PTA	AVSD	LAA	N/A	L(St/Sp/P)	Oligo

***Mosmo*$^{-/-}$ embryos with 3 Day and 4 Day vismodegib treatment**

Embryo ID	Vismo (Days)	Body situs	Heart	VA Alignment	Septal Defects	Aortic Arch	Lung Situs	Abdomen Situs	Digitation
1	3	HTX	Dex	DORV	AVSD	RAA	3R:3L:1M	L(St/Sp/P)	Normal/Oligo/PDD
2	3	HTX	Lev	DORV	AVSD	LAA	3R:3L:1M	L(St/Sp/P)	PDD
3	3	HTX	Lev	DORV	AVSD	LAA	3R:3L:1M	R(St/Sp/P)	PDD
4	3	HTX	Lev	TGA	AVSD	LAA	1R:4L	L(St/Sp/P)	Normal/PDD
5	3	HTX	Lev	TGA	AVSD	LAA	1R:4L	L(St/Sp/P)	Normal/PDD
6	3	HTX	Lev	TGA	AVSD	LAA	1R:4L	L(St/Sp/P)	Normal/PDD
7	4	HTX	Lev	DORV	AVSD	LAA	1R:4L	L(St/Sp/P)	Normal/PDD
8	4	HTX	Lev	DORV	AVSD	RAA	3R:3L:1M	L(St/Sp/P)	Normal/PDD

Lung situs could not be determined (N/A) in vismodegib treated control embryos due to cystic and hypoplastic lungs. AVSD, atrioventricular septal defect; CC, concordant ventriculoarterial alignment; Dex, dextrocardia; DORV, double outlet right ventricle; HAA, hypoplastic arch; HTA, hypoplastic transverse heart; HTX, heterotaxy; IAA, interrupted aortic arch; LAA, left aortic arch; Lev, levocardia; L(St/Sp/P), Left sided stomach, spleen, pancreas; Mes, mesocardia; N/A: no digit data collected; NML, normal digit pattern; Oligo, oligodactyly; PDD, preaxial digit duplication; PTA, Persistent truncus arteriosus; RAA, right aortic arch; RPI, right pulmonary isomerism; R(St/Sp/P), Right sided stomach, spleen, pancreas; SIT, situs inversus; SS, situs solitus; SymLiv, symmetric liver; TGA, transposition of the great arteries; VA, ventriculoarterial; VSD, ventricular septal defect; VSD(Ao), ventricular septal defect located below the aorta; 1L, one lung lobe on left side; 4R, 4 lung lobes on right side.

Development • Supplementary information

Table S5. Related to Figure 5: Genotypes of live embryos derived from *Mosmo*$^{+/-}$ intercrosses treated with vehicle or vismodegib.

Treatment	Age	*Mosmo*$^{+/+}$	*Mosmo*$^{+/-}$	*Mosmo*$^{-/-}$	Total	Chi square p-value
Expected		*25%*	*50%*	*25%*		
Vehicle treated	e14.5	8 (21.6%)	28 (75.7%)	1 (2.7%)	n = 37	p = 0.002024
Vismodegib treated	e14.5	8 (17.4%)	22 (47.8%)	16 (34.8%)	n = 46	p = 0.238175

Significant deviation from the expected Mendelian ratio of 1:2:1 was calculated using the chi-square test (ns>0.05 and **P<0.01).

Development • Supplementary information
